# Identification of compounds from *Origanum compactum* and *Origanum elongatum* using HPLC/UV-ESI-MS and comparative analysis of their antioxidant, antimicrobial, anticoagulant, and antidiabetic properties

**DOI:** 10.1016/j.jsps.2024.102184

**Published:** 2024-10-02

**Authors:** Omkulthom Al Kamaly, Aziz Drioiche, Firdaous Remok, Soukaina Saidi, Ahde El Imache, Fadoua El Makhoukhi, Bshra A. Alsfouk, Touriya Zair

**Affiliations:** a Department of Pharmaceutical Sciences, College of Pharmacy, Princess Nourah bint Abdulrahman. University, P.O. Box 84428, Riyadh 11671, Saudi Arabia; b Research Team of Chemistry of Bioactive Molecules and the Environment, Laboratory of Innovative Materials and Biotechnology of Natural Resources, Faculty of Sciences, Moulay Ismaïl University, B.P. 11201 Zitoune, Meknes 50070, Morocco; c Higher Institute of Nursing Professions and Health Techniques of Fez, Regional Health Directorate Fez-Meknes, EL Ghassani Hospital, 30000 Fez, Morocco; d Laboratory of Innovative Technologies, Process Engineering Department, Higher School of Technology Fez, USMBA, Fes, Morocco

**Keywords:** *Origanum compactum*, *Origanum elongatum*, Lithospermic acid, Salvianolic acid C, Rosmarinic acid, Antioxidant, Antimicrobial, Anticoagulant, Antidiabetic

## Abstract

The aim was to assess the phytochemical composition, phenolic component levels, and biological properties of the flowering tops of *Origanum compactum* and *Origanum elongatum*. The study employed phytochemical assays, spectrophotometric techniques for quantitative analysis of polyphenols, flavonoids, and tannins, and compound identification using HPLC/UV-ESI-MS. The antimicrobial, antioxidant, anticoagulant, and antidiabetic properties were examined both *in vitro* and *in vivo*. The results showed that the *O. compactum* extract had significantly high levels of total polyphenols, measuring 47.368 mg gallic acid equivalents per gram, and flavonoids, measuring 14.839 mg quercetin equivalents per gram. The phytochemical examination of *O. compactum* revealed that lithospermic acid accounted for 36.82 % of the chemicals detected, followed by salvianolic acid C at 12.57 % and ros-marinic acid at 6.01 %. The main constituents of *O. elongatum* are salvianolic acid C (14.46 %), luteolin-3-O-glucuronide (13.51 %), salvianolic acid B (12.24 %), rosmarinic acid (7.83 %), and rutin (6.18 %). The results demonstrated different levels of effectiveness against the investigated microorganisms, with the extract from *O. compactum* exhibiting better activity, particularly against Gram-negative bacteria, certain yeasts, and the fungus Aspergillus niger. The aqueous extracts of both *Origanum* species demonstrate significant antioxidant activity. *O. compactum* has a higher total antioxidant capacity (IC_50_ of 35.083 μg/mL) compared to *O. elongatum* (IC_50_ of 77.080 μg/mL). However, *O. elongatum* has a higher reducing power (35.697 μg/mL) compared to *O. compactum* (42.563 μg/mL). *In vivo* evaluations revealed that the aqueous extracts of *O. compactum* and *O. elongatum* possess significant antihyperglycemic and anticoagulant properties. The extracts demonstrated a marked reduction in blood glucose levels during the oral glucose tolerance test (OGTT) in Wistar rats and effectively prolonged both prothrombin time (PT) and activated partial thromboplastin time (aPTT), highlighting their ability to inhibit coagulation pathways. Moreover, their comparable efficacy to standard antihyperglycemic medications and absence of severe toxicity, even at high doses, underscore their therapeutic potential for safe and effective treatment applications. Between the two species, *O. compactum* exhibited superior efficacy in key biological activities such as antioxidant, antimicrobial, and anticoagulant properties, making it a strong candidate for therapeutic applications. This study underscores the value of *Origanum* species as a rich source of bioactive compounds, offering significant potential in pharmaceuticals, nutraceuticals, and agri-food industries. The findings pave the way for further exploration of their diverse applications.

## 1 Introduction

Reactive oxygen species (ROS) generation that is excessive can lead to oxidative stress, which is a primary pathogenic mechanism that seriously damages vital biomolecules including proteins, lipids, and DNA. Complex and incapacitating illnesses such as cancer, diabetes, cardiovascular disease, and neurodegenerative disorders are brought on by these damages (Bhattacharyya et al., 2014, Vona et al., 2021). Even while ROS are typical results of cellular metabolism, they have the potential to outweigh an organism's antioxidant defenses, resulting in severe molecular alterations that eventually cause tissue and cell degeneration (Olufunmilayo et al., 2023). Although various therapeutic approaches exist, the urgency for novel, safer, and more effective treatments remains due to the adverse side effects associated with current therapies.

The selection of antioxidant, antimicrobial, anticoagulant, and antidiabetic activities for evaluation is based on their direct relevance to combating oxidative stress-related diseases and infections, which are key contributors to chronic illnesses such as cancer, cardiovascular disease, and diabetes. These biological activities were chosen because they provide a comprehensive understanding of the therapeutic potential of the plant's bioactive compounds, such as polyphenols and flavonoids, which have been linked to antioxidant, antimicrobial, and anticoagulant properties. Additionally, their ability to influence metabolic pathways, including glucose regulation, supports the investigation of their antidiabetic potential.

In light of these challenges, significant research has focused on bioactive compounds derived from natural plant sources, especially aromatic and medicinal plants rich in various classes of chemical compounds, including polyphenols, terpenic compounds, and essential oils. These bioactive components are known for their antibacterial, anti-inflammatory, and antioxidant properties, offering a promising path for developing alternative therapeutic agents. For instance, carvacrol and thymol, primary constituents of oregano essential oils, have exhibited significant antibacterial and antioxidant properties (Chroho et al., 2024, Hajibonabi et al., 2023). Furthermore, these plant-derived compounds, such as polyphenols and flavonoids, are increasingly being investigated for their potential role in combating oxidative stress-related diseases.

There is strong scientific support for the use of medicinal plants in the treatment of chronic illnesses. For instance, turmeric is known for its anti-inflammatory and anti-cancer benefits (Cai et al., 2016), while rosemary is well-known for its hepatoprotective and cardioprotective qualities (Rahbardar et al., 2022). Furthermore, the investigation of plant biodiversity in particular areas, like Morocco, creates new opportunities for the finding of unique bioactive compounds.

Humanity has traditionally used the benefits of plants to maintain and enhance health and wellbeing. Medicinal plants have been used in medicine from the time of ancient civilizations like Egypt and Greece. The use of plants to treat a variety of ailments and enhance quality of life has been made possible by this empirical knowledge that has been passed down from generation to generation.

Morocco benefits from an abundant and diverse flora, encompassing approximately 7,000 species, subspecies, and varieties distributed across 940 genera and 135 families (Cherrat et al., 2024). This floristic diversity is favored by a variety of ecosystems, ranging from desert areas to the Atlas Mountains, creating unique ecological niches that harbor considerable plant wealth. Among these plants, many species possess recognized medicinal properties and have been used for centuries in traditional Moroccan medicine.

The Fez-Meknes region, characterized by its diverse ecosystems such as forests, plains, and mountainous areas, presents an opportune environment for the exploration and identification of new bioactive molecules of potential interest to the agri-food and pharmaceutical sectors. Among the region's most notable aromatic and medicinal plants is oregano, a member of the Lamiaceae family. Due to its significant economic and social impact, the conservation of this species has become imperative.

The scientific community has thoroughly investigated the genus Origanum, which is part of the Lamiaceae family and encompasses 38 species, 6 subspecies, and 17 hybrids, predominantly distributed in the Mediterranean, Irano-Turanian, and Euro-Siberian regions, and is especially appreciated for its essential oils (Bachar et al., 2021). Because of their therapeutic qualities, these plants are used as natural antiseptics, to treat digestive issues, and to cure respiratory infections (Murad et al., 2021, Schuetz, 2016). Moreover, research has demonstrated that oregano extracts have strong antioxidant qualities (Amarowicz et al., 2009, Cervato et al., 2000), which makes them a viable option for the creation of novel therapeutic agents.

The traditional uses of aromatic and therapeutic plants from the Fez-Meknes area are not the only ways in which they are valued. To fully explore their potential in contemporary applications, such as the development of novel medications, nutritional supplements, and natural cosmetics, research and development projects are now under progress. In addition to improving the health and well-being of the local populace, these initiatives support regional economic growth and biodiversity preservation.

Many types of oregano, known locally as “Za'atar” or “Zwi” in Berber, are well known for their therapeutic benefits in Morocco (Tagnaout et al., 2023). These plants have a big role in traditional Moroccan medicine, which uses them to cure a lot of different illnesses and ailments. It is advised to use these plants' decoctions to cure a range of diseases, including colitis, diarrhea, pulmonary and bronchial problems, stomach hyperacidity, and gastrointestinal disorders (Hachi et al., 2022, Hoffmann, 2003, Lokare et al., 2022, Midaoui et al., 2011). These age-old treatments are handed down from generation to generation and have a strong cultural foundation in the area.

In specific regions, such as the Middle Atlas, oregano is also used to treat liver ailments (El-Ghazouani et al., 2024). Local populations often resort to infusions and decoctions of this plant to alleviate various ailments, drawing on ancestral knowledge accumulated over centuries (El-Ghazouani et al., 2024). Indeed, the use of medicinal plants, including oregano, is an integral part of the traditional Moroccan pharmacopeia (Ajebli and Eddouks, 2023), which remains an essential pillar of primary healthcare, particularly in rural areas where access to modern medical care is limited.

Some of the traditional applications of oregano have been supported by modern scientific studies that have revealed the plant's many biological qualities. These herbs have antibacterial, anti-inflammatory, antioxidant, anticancer, antifungal, antiviral, and anti-leishmanial properties in addition to their medicinal benefits (Baser, 2002, Marrelli et al., 2018, Ortega-Ramirez et al., 2016, Singh and Sharma, 2020). The scientific community has recognized these qualities, which has increased interest in these plants for potential medical uses.

The uniqueness of this study lies in its focus on the Fez-Meknes region of Morocco, a biodiversity hotspot with diverse ecosystems-forests, plains, and mountainous areas-that present an ideal environment for discovering novel bioactive molecules. Despite the vast scientific evidence supporting the therapeutic potential of medicinal plants, there remains a gap in knowledge regarding the specific chemical components and their mechanisms of action in certain endemic species. This study specifically aims to contribute to filling this gap by conducting a detailed chemical and biological characterization of *O. compactum* and *O. elongatum*, two species of oregano known locally as “Za'atar” or “Zwi” in Berber.

Therefore, the purpose of this study is to elucidate the chemical profiles of O. compactum and *O. elongatum* using advanced analytical techniques such as high-performance liquid chromatography coupled with electrospray ionization mass spectrometry (HPLC/UV-ESI-MS). Additionally, the study design includes a comprehensive evaluation of their antioxidant, antimicrobial, anticoagulant, and antidiabetic activities, providing a comparative analysis of their therapeutic potential. These stages involve the isolation and quantification of key bioactive compounds, followed by *in vitro* assays to determine their biological efficacy against targeted diseases.

This research not only emphasizes the importance of conserving these species due to their significant economic and social impact but also explores their potential applications in developing new medications, nutritional supplements, and natural cosmetics. By providing a deeper understanding of the phytochemical composition and therapeutic properties of these oregano species, this study aims to offer novel insights that could lead to innovative applications in phytomedicine, thus enriching the existing scientific literature and opening new perspectives in natural product research.

## 2 Materials and methods

### 2.1 Chemicals and reagents

All chemicals and solvents used in this investigation were of analytical quality and procured from Sigma Aldrich, Morocco, except for those employed in coagulation assays, which were acquired from Diagnostica Stago, France. Ethanol, acetic acid, and formic acid functioned as solvents for the preparation of several solutions, including the DPPH solution used in antioxidant activity experiments. Hydrochloric acid (HCl) was used for the quantification of condensed tannins, whereas nitric acid (HNO_3_) was applied for the examination of heavy metals using Inductively Coupled Plasma Atomic Emission Spectrometry (ICP-AES). Ammonium molybdate was used to assess Total Antioxidant Capacity (TAC), whilst aluminum chloride (AlCl_3_) was utilized for the colorimetric determination of flavonoids. Sodium hydroxide (NaOH), potassium phosphate, and sodium phosphate were used in the formulation of several buffers for enzymatic tests. Additional reagents included potassium iodate (KIO_3_) for tannin measurement, 3,5-dinitrosalicylic acid (DNSA) for terminating enzymatic processes in antidiabetic experiments, and calcium chloride (CaCl_2_) for commencing coagulation tests.

### 2.2 Study area

**Fig. 1:**
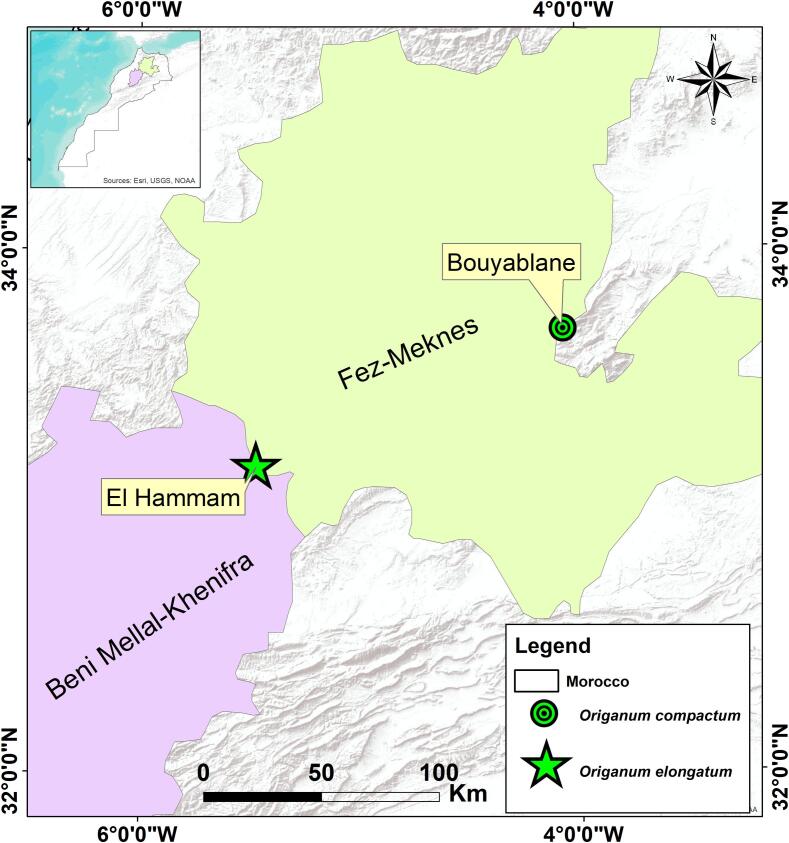
Harvesting sites for *Origanum* species studied.

The flowering tops of both oregano species were harvested from two different mountainous sites: Bouyablane for *O. compactum* and El Hamam de Khenifra for *O. elongatum* (Fig. 1).

### 2.3 Plant material

The *O. compactum* and *O. elongatum* specimens studied were harvested in July 2022 during the flowering phase. The research largely employed the aerial portions of the plant, including the leaves and flowering tops, which are recognized for their high quantities of phenolic chemicals. The components were meticulously dehydrated in a darkened environment for around 10 days to retain their bioactive properties. The species was botanically recognized by Professor Hamid Khamar at the Floristics Laboratory of the Scientific Institute of Rabat, assuring precise species identification.

### 2.4 Microbial materials

The antibacterial efficacy of the aqueous extract was evaluated against twenty-four bacterial and eight fungal strains (Table 1). These particular microbes are harmful and well-known for their strong resilience, ability to invade, and toxicity to humans. These organisms are often associated with many diseases in Morocco, presenting a considerable challenge for clinical diagnosis and treatment. The strains were acquired from the hospital environment of Mohamed V-Meknes Provincial Hospital. The strains were preserved in a 20 % glycerol solution at −80 °C. They were then revived in Mueller-Hinton and Sabouraud broths, and subsequently cultivated again before use.

**Table 1:** Compilation of Evaluated Bacterial and Fungal Strains Alongside Their Corresponding References.

**Strains**		**Abbreviations**	**References**
**Gram-positive cocci**	*Staphyloccocus epidermidis*	*S. epidermidis*	5994
	*Staphyloccocus aureus BLACT*	*S. aureus BLACT*	4IH2510
	*Staphyloccocus aureus STAIML/MRS/mecA/HLMUP/BLACT*	*S. aureus STAIML/MRS/mecA/HLMUP/BLACT*	2DT2220
	*Streptococcus acidominimus*	*S. acidominimus*	7DT2108
	*Streptococcus group D*	*S. group D*	3EU9286
	*Streptococcus agalactiae*	*S. agalactiae*	7DT1887
	*Streptococcus porcinus*	*S. porcinus*	2EU9285
	*Enterococcus faecalis*	*E. faecalis*	2CQ9355
	*Enterococcuss faecium*	*E. faecium*	13EU7181

**Gram-negative bacilli**	*Acinetobacter baumannii*	*A. baumannii*	7DT2404
	*Escherichia coli*	*E. coli*	3DT1938
	*Escherichia coli ESBL*	*E. coli ESBL*	2DT2057
	*Enterobacter aerogenes*	*E. aerogenes*	07CQ164
	*Enterobacter cloacae*	*E. cloacae*	02EV317
	*Citrobacter koseri*	*C. koseri*	3DT2151
	*Klebsiella pneumonie* ssp. *pneumonie*	*K. pneumonie*	3DT1823
	*Proteus mirabilis*	*P. mirabilis*	2DS5461
	*Pseudomonas aerogenosa*	*P. aerogenosa*	2DT2138
	*Pseudomonas fluorescence*	*P. fluorescence*	5442
	*Pseudomonas putida*	*P. putida*	2DT2140
	*Serratia marcescens*	*S. marcescens*	375BR6
	*Salmonella* sp.	*Salmonella* sp.	2CG5132
	*Shigella* sp.	*Shigella* sp.	7DS1513
	*Yersinia enterocolitica*	*Y. enterocolitica*	ATCC27729

**Yeasts**	*Candida albicans*	*C. albicans*	Ca
	*Candida kefyr*	*C. kefyr*	Cky
	*Candida krusei*	*C. krusei*	Ckr
	*Candida parapsilosis*	*C. parapsilosis*	Cpa
	*Candida tropicalis*	*C. tropicalis*	Ct
	*Candida dubliniensis*	*C. dubliniensis*	Cd
	*Saccharomyces cerevisiae*	*S. cerevisiae*	Sacc

**Fungi**	*Aspergillus niger*	*A. niger*	AspN

### 2.5 Selection of animals for research

Acute toxicity studies were conducted on male and female albino mice (20–25 g, 6–8 weeks old), and the antidiabetic effects of a substance were investigated in male and female Wistar rats (200–250 g, 8–10 weeks old). All animals used in this study were bred in-house at the animal facility of the Department of Biology, Faculty of Sciences, Dhar El Mahraz, Fez. The animals were maintained under standard laboratory conditions, with a 12-hour light–dark cycle at a temperature of 22 ± 2 °C, and had unrestricted access to food and water. Prior to the experiments, all animals were acclimatized to these conditions for at least one week. All procedures involving the care, use, and handling of animals were carried out in accordance with the Guidelines for the Care and Use of Laboratory Animals issued by the National Institutes of Health (NIH) and approved by the institutional animal ethics committee (02/2019/LBEAS (Albus, 2012).

### 2.6 Phytochemical screening

This qualitative investigation involved solubility testing, precipitation, turbidity assessment, detection of color changes, and UV light inspection to identify chemical families. The phytochemical analysis utilized the flowering tops of the oregano species under study. Plant samples were dried and finely powdered for analysis, employing methods described by Dohou et al., Judith, Mezzoug et al., Bekro et al., Bruneton, and N'Guessan et al. (Bekro et al., 2007, Bruneton, 2009, Dohou et al., 2003, Judith, 2005, Mezzoug et al., 2007, N’Guessan et al., 2009) to determine the presence of various chemical groups.

### 2.7 Investigation of phenolic compounds

#### 2.7.1 Extraction procedure for phenolic compounds

The decoction was used to extract phenolic chemicals. A 30 g sample was mixed with 600 ml of distilled water and heated to 80 °C for 1 h. After the boiling operation, the solution was allowed to stand undisturbed for 5 min before being filtered under reduced pressure. The concentrated extract was then dried in an oven at 70 °C. The resultant product, as a powdered substance, was meticulously gathered in a glass flask and stored for future use.

To quantify phenolic components and evaluate biological and pharmacological activity, the dried extract was reconstituted in sterile distilled water. The extract used for these experiments was a decoction, which is an aqueous extract. No other solvents or reagents were necessary during this step, save for sterile distilled water to dissolve the dry extract for the next analyses.

#### 2.7.2 Quantification of total polyphenols

The Folin–Ciocalteu method, as described by Singleton and Rossi (Singleton and Rossi, 1965), was used to quantify the total polyphenol content in the plant extracts. This technique entails the oxidation of the polyphenols, followed by the production of color. The Folin–Ciocalteu reagent, composed of phosphotungstic (H_3_PW_12_O_40_) and phosphomolybdic (H_3_PMo_12_O_40_) acids, undergoes a reaction to produce blue tungsten oxides (W_8_O_23_) and molybdenum oxides (Mo_8_O_3_). The oxides were later examined using colorimetry at a wavelength of 760 nm. Absorbance measurements were conducted using a UV mini-1240 spectrophotometer, comparing the samples to a blank composed of the reaction mixture devoid of the extract. Gallic acid was used as the positive control at various doses beginning from 50 µg/mL. A calibration curve was produced under identical experimental circumstances. The findings are quantified in milligrams of gallic acid equivalents per gram of extract (mg GAE/g), derived from the calibration curve equation (Y = ax + b). Each test was conducted thrice to guarantee precision.

#### 2.7.3 Quantification of flavonoids

The measurement of flavonoid concentration was conducted using the colorimetric technique with aluminum trichloride, following the protocols described by Djeridane (Djeridane et al., 2006), Hung (Hung et al., 2009), and their collaborators. The compound aluminum chloride (AlCl_3_) undergoes a chemical reaction with the hydroxyl groups (OH) present in flavonoids, resulting in the formation of a complex. The flavonoid content was measured using UV spectroscopy at a wavelength of 433 nm. Quercetin, a well-known flavonoid, was used as a reference under identical analytical circumstances, with concentrations varying from 5 to 30 µg/mL. A calibration curve (Y = ax + b) was created using quercetin standards to ascertain the flavonoid content in milligrams per gram of extract (mg/g). The experiment was conducted in duplicate to ensure precision.

#### 2.7.4 Quantification of condensed tannins

The vanillin test (Price et al., 1978) was employed to quantify the amounts of condensed tannins. Concisely, various volumes of a solution containing (+)-catechin (2 mg/mL) were added to 3 mL of a vanillin/methanol solution (4 %; w/v). The liquid was stirred by hand, and then 1.5 mL of strong hydrochloric acid was added to each sample. The resultant mixes had a 20-minute reaction at ambient temperature. Absorbance was quantified at a wavelength of 499 nm with a UV–visible spectrophotometer, in contrast to a blank sample. During the development of the calibration curve for the measurement of condensed tannins, catechin was used as a substitute for the samples. This curve facilitated the quantification of tannin content, articulated as milligrams of catechin equivalents per gram of dry weight.

#### 2.7.5 Quantification of hydrolyzable tannins

A modified version of the Willis and Allen technique (Willis, 1998) was used to quantify hydrolyzable tannins. A vigorous amalgamation of 10 µL of the extract with 5 mL of a 2.5 % KIO_3_ solution was conducted for ten seconds. Under ideal reaction circumstances, the extract attained its maximum absorbance in 2 min, whereas the conventional tannic acid solution reached its peak absorbance in 4 min. Absorbance was quantified at a wavelength of 550 nm with a UV–visible spectrophotometer. The results were measured in milligrams of tannic acid per gram of plant material that had been dried. An 11-point calibration curve was constructed using various amounts of tannic acid, ranging from 100 to 2000 µg/mL.

#### 2.7.6 HPLC/UV ESI-MS analysis of decoctions from studied Origanum species

The phenolic components in the extracts were examined using high-performance liquid chromatography combined with Q Exactive Plus mass spectrometry, which utilizes electrospray ionization (HPLC/UV-ESI-MS). The study employed an UltiMate 3000 HPLC system (Thermo Fisher Scientific, Sunnyvale, CA, USA) that included a sample changer to keep samples at a temperature of 5 °C. A reverse phase C18 column (250 × 4 mm, 5 μm, Lichro CART, Lichrospher, Merck, Darmstadt, Germany) was used at a consistent column temperature of 40 °C. The mobile phase, degassed by ultrasonic treatment, included solvent A (0.1 % formic acid in water, v/v) and solvent B (0.1 % formic acid in acetonitrile, v/v). The gradient started with 2 % solvent B at 0 min, incrementally rising to 30 % by 20 min, to 95 % by 25 min, and thereafter declining to 2 % at 26 min, where it stabilized until 30 min. The flow rate was 1 ml per minute, and 20 microliter injections were generated.

Detection was conducted using a Maxis Impact HD apparatus (Bruker Daltonik, Bremen, Germany) in MS/MS mode with broadband collision-induced dissociation (bbCID) subsequent to negative electrospray ionization. A L-2455 diode array detector (Merck-Hitachi, Darmstadt, Germany) was used for UV detection, scanning wavelengths from 190 to 600 nm, with targeted data gathering at 280 nm, 320 nm, and 360 nm. The experimental configuration included a capillary voltage of 3000 V, a drying gas temperature of 200 °C, a dry gas flow rate of 8 L/min, a nebulizing gas pressure of 2 bar, and an offset plate voltage of −500 V, using nitrogen as the desolvation and nebulizer gas. Mass spectrometry (MS) data were acquired throughout the mass-to-charge ratio (*m*/*z*) range of 100 to 1500. Data collection and analysis were performed with the Thermo Scientific™ Chromeleon™ 7.2 Chromatography Data System (CDS) software, and the eluted chemicals were evaluated by their mass spectra.

### 2.8 Analysis of heavy metals: inductively coupled plasma atomic emission spectrometry (ICP-AES)

The study examined many heavy metals, specifically arsenic (As), cadmium (Cd), chromium (Cr), iron (Fe), lead (Pb), antimony (Sb), and titanium (Ti). Every metal has its own set of contamination standards, some of which are very strict. These restrictions exclude drugs that include main components that are known to accumulate substantial quantities of cadmium. The preferred method for analyzing the major elements (arsenic, cadmium, chromium, iron, lead, antimony, and titanium) is the mineralization approach proposed by AFNOR in 1999, which involves the use of aqua regia, a combination of nitric and hydrochloric acids. This approach enables the utilization of bigger sample sizes to improve the representativeness of the sample. The method entailed combining 0.1 g of finely pulverized plant material with 3 mL of aqua regia solution (consisting of 1 mL of 99 % nitric acid and 2 mL of 37 % hydrochloric acid), and thereafter subjecting it to reflux at a temperature of 200 °C for a duration of two hours. Following the process of cooling and sedimentation, the liquid that settled at the top was separated and filtered using a membrane with a pore size of 0.45 µm. The filtered liquid was then mixed with distilled water to reach a final volume of 15 mL. The ICP-AES equipment (Ultima 2 Jobin Yvon) at the UATRS laboratory (Technical Support Unit for Scientific Research) of CNRST in Rabat (Skujins, 1998) was used to measure the amounts of heavy metals.

### 2.9 Antioxidant activities

#### 2.9.1 Test assessment of antiradical activity using the DPPH• test

The assessment of antiradical activity relied on the capacity of an antioxidant (phenolic molecule) to donate a single electron to the synthetic radical DPPH• (2,2-diphenyl-1-picrylhydrazyl), leading to its decrease from purple to yellow, therefore stabilizing it (Brand-Williams et al., 1995). The experiment used a UV–visible spectrophotometer, measuring at a wavelength of 515 nm. A DPPH• solution with a concentration of 6 × 10^−5^ M was created by dissolving 2.4 mg of DPPH• in 100 mL of ethanol. The extract samples were dissolved in pure ethanol. The process included combining 200 µL of either the extract (sample) or the reference antioxidant (ascorbic acid) at different concentrations with 2.8 mL of the produced DPPH• solution. Following a 30-minute incubation at ambient temperature in the absence of light, the absorbance of the reaction mixture was assessed at 515 nm, using pure ethanol as the blank control. The DPPH• solution without extract was used as the negative control. The calculation of percent inhibition was performed using formula (1) (Tagnaout et al., 2016), which was based on the absorbance data.

(1) $$\%AA=\frac{Abs\;control\;-\;Abs\;sample}{Abs\;control}\times 100$$

where: % AA is the proportion of antiradical activity; Abs control refers to the absorbance of the blank, which is the optical density of the solution containing DPPH• and ethanol. Abs sample: The absorbance of the test chemical (extracts).

#### 2.9.2 Ferric reducing antioxidant power (FRAP) assay

We utilized Oyaizu's 1986 technique (Oyaizu, 1986) to assess the iron-reducing capability of our extracts. This involved quantifying the conversion of Fe^3+^ to Fe^2+^ in the K_3_Fe(CN)_6_ complex. To evaluate the antioxidant activity utilizing the FRAP approach, samples obtained from different extracts were subjected to the following process. A fraction of the extract adhered to the same methodology outlined for the preceding samples. The spectrophotometer was calibrated using pure water, and absorbance measurements were recorded at a wavelength of 700 nm, using a blank as the reference standard. Ascorbic acid served as the positive control, evaluated under identical circumstances to the extract samples. An elevation in absorbance correlated with an augmented reducing power of the extracts.

#### 2.9.3 Determination of total antioxidant capacity (TAC)

The total antioxidant capacity (TAC) of the extracts was evaluated using the phosphomolybdenum technique established by Khiya (Khiya et al., 2021). This technique entails the reduction of molybdenum Mo (VI) to molybdenum Mo (V) in the presence of the extract. Molybdenum first occurs as molybdate ions (MoO_4_^2−^), which are reduced in acidic circumstances to yield a green phosphate/Mo(V) complex. The reagent solution used in this experiment included 0.3 mL of 0.6 M sulfuric acid, 28 mM sodium phosphate, and 4 mM ammonium molybdate, which was incorporated into each extract. The tubes were sealed and incubated at 95 °C for 90 min. Following chilling, the absorbance of the solutions was assessed at 695 nm, with a blank solution undergoing the same conditions as the sample. A calibration curve was created with ascorbic acid standards at different concentrations. The TAC values were quantified as milligrams of ascorbic acid equivalents per gram of crude extract (mg AAE/g).

### 2.10 Determination of minimum inhibitory concentration (MIC), minimum bactericidal concentration (MBC), and minimum fungicidal concentration (MFC)

The minimum inhibitory concentration (MIC) was measured by employing 96-well microplates and the conventional microdilution method (Balouiri et al., 2016). The term Minimum Inhibitory Concentration (MIC) refers to the smallest amount of an antibiotic substance, such as an extract, that is needed to totally prevent the development of a microbe within a specific length of time. This is determined by observing the absence of visible growth without the use of magnification. The decoctions from the two examined *Origanum* species were made using a 10 % DMSO solution, resulting in a concentration range of 5 to 0.93 × 10^−2^ mg/mL for each decoction. The dilutions were created by incorporating 100 µL of each concentration into Sabouraud broth for fungus and Mueller-Hinton medium for bacteria. Subsequently, 100 µL of microbial inoculum, with concentrations of 10^6^ CFU/mL for bacteria and 10^4^ CFU/mL for fungus, was included in the dilution series. Following a 24-hour incubation at 37 °C, 10 µL of resazurin was added to each well to evaluate bacterial growth, shown by a color transition from purple to pink after an additional two-hour incubation at the same temperature, indicating microbial activity. The minimum inhibitory concentration (MIC) is defined as the lowest concentration at which no color change is seen in resazurin. The eleventh and twelfth wells functioned as controls for growth and sterility, respectively. The examination was administered twice for precision. Furthermore, 250 mg of Terbinafine, a prevalent antifungal compound, was solubilized in 2 mL of 10 % DMSO after its pulverization into a powder. The Minimum Bactericidal Concentration (MBC) and Minimum Fungicidal Concentration (MFC) were established by transferring 10 µL from wells exhibiting no visible growth onto Mueller-Hinton agar for bacteria or Sabouraud broth for fungi, followed by a 24-hour incubation at 37 °C. The MBC and MFC were determined to be the minimal concentrations that achieved a 99.99 % decrease in colony-forming units per milliliter (CFU/mL) relative to the control. Furthermore, the MBC/MIC or MFC/MIC ratio was determined for each extract to evaluate its antibacterial efficacy. A ratio less than 4 indicates the presence of bactericidal or fungicidal activity, whilst a ratio greater than 4 reveals the presence of bacteriostatic or fungistatic effects (Bissel et al., 2014).

### 2.11 Anticoagulant activity

The anticoagulant effect was assessed using chronometric coagulation tests, namely prothrombin time (PT) and activated partial thromboplastin time (aPTT), following the methodology outlined by Hmidani et al. (Hmidani et al., 2019). Decoctions were formulated and evaluated for their capacity as anticoagulant agents. The coagulation tests utilized values of 11.500, 5.750, 2.875, 1.438, 0.719, 0.359, and 0.179 mg/mL. Trisodium citrate tubes with a concentration of 3.8 % were used to collect blood samples. The samples were promptly centrifuged at a speed of 25,000 rpm for a duration of 10 min. This process was done to separate and combine the plasma, which was thereafter kept at a temperature of −10 °C until it was needed. To assess aPTT, 50 µL of citrated normal plasma was mixed with 50 µL of the plant extract solution and incubated at 37 °C for 10 min. Subsequently, 100 µL of PTT reagent (CKPREST®) was introduced, and the mixture was incubated at 37 °C for 5 min. The coagulation process started with the addition of 100 µL of a 25 mmol/L CaCl_2_ solution, and the clotting time was documented. In the PT assessment, 50 µL of citrated normal plasma pool was combined with 50 µL of the plant extract solution and incubated for 10 min. Subsequently, 200 µL of Neoplastin® Cl reagent was added, followed by an additional 10-minute incubation at 37 °C. The coagulation time was assessed. The anticoagulant efficacy of several aqueous plant extracts was evaluated in seconds at varying concentrations, with the assay conducted six times with an automated coagulometer, the MC4Plus MERLIN Medical®.

### 2.12 Antidiabetic activity

#### 2.12.1 Evaluation of aqueous extracts' inhibitory effect on pancreatic α-amylase activity, *in vitro*

The aqueous extract's inhibitory effect on α-amylase enzymatic activity was evaluated according to the procedure established by Daoudi et al. (Daoudi et al., 2020). A phosphate buffer solution was combined with either 200 µL of the aqueous extract solution or 200 µL of acarbose solution, which served as a positive control. All tubes, save the blank, were augmented with 200 µL of the enzyme solution, whilst the blank was allocated 200 µL of phosphate buffer. The tubes were then pre-incubated for 10 min at 37 °C. Afterward, 200 µL of starch solution was added to each tube. Subsequently, the reaction mixture was subjected to incubation at a temperature of 37 °C for a duration of 15 min. To stop the enzymatic process, 600 µL of DNSA solution was introduced to each tube and then incubated in boiling water for 8 min. Subsequently, the tubes were chilled in an ice water bath, and 1 mL of distilled water was introduced to each tube. Absorbance was quantified at 540 nm using a spectrophotometer, with the blank comprising a buffer solution instead of the enzyme solution. The % inhibition was then computed using Eq. (2).

(2) $$\%Inhibition=\frac{A\;control\;-\;A\;sample}{A\;control}\;\times \;100$$

The control pertains to the absorbance recorded for enzymatic activity without the presence of any inhibitor. The sample demonstrates absorbance linked to enzymatic activity with the introduction of either the extract or acarbose.

#### 2.12.2 Investigation into the inhibitory impact of aqueous extracts on α-glucosidase activity, conducted *in vitro*

The inhibitory action of the extract on α-glucosidase was assessed using the pNPG substrate, according to a modified protocol outlined by Chatsumpun et al. (Chatsumpun et al., 2017). The extracts were evaluated within a concentration range of 0.488 to 100 µg/mL. Samples were produced in a 5 % DMSO solution, and the α-glucosidase enzyme was dissolved in phosphate buffer at pH 6.8. The solvent control consisted of 5 % DMSO, whereas acarbose functioned as the positive control. Forty microliters of α-glucosidase enzyme (0.1 units/mL) and ten microliters of each sample were introduced onto a 96-well plate, thereafter undergoing pre-incubation at 37 °C for ten minutes. Thereafter, 50 µL of pNPG (1 mM) was added, and the mixture was incubated at 37 °C for 20 min. The reaction was concluded by the addition of 100 µL of 0.1 M sodium carbonate solution. The optical density of the combination was assessed at 405 nm using a microplate reader. The inhibition percentage of α-glucosidase was determined using Eq. (2).

#### 2.12.3 Acute toxicity study

This experiment aimed to evaluate the immediate harmful effects of orally administering decoctions of *O. elongatum* and *O. compactum*, using the usual methods of exposure under normal conditions. The study was done following the rules set by the Organization for Economic Cooperation and Development (OECD) (Weng et al., 2021). The chosen items were picked for their pharmacological study. The dosages provided were 0.5, 1, and 2 g/kg.

Two cohorts of albino mice, with a weight range of 20 to 35 g during 14 h of fasting, were randomly allocated into four groups. Each group consisted of six mice, with an equal mix of males and females. The control group was administered a dosage of 10 mL/kg of distilled water. Group (1) was administered an aqueous extract at a dosage of 0.5 g per kilogram, Group (2) at a dosage of 1 g per kilogram, and Group 3 at a dosage of 2 g per kilogram. Before the experiment, the mice were weighed and given a single dosage of the aqueous extract. They were thereafter examined without interruption for a duration of 10 h in order to detect any indications of acute toxicity. Observations were conducted daily for the next 14 days to evaluate for any potential signs of damage, such as alterations in physical state and behavior.

#### 2.12.4 Investigation of the antihyperglycemic activity of aqueous extracts from the studied Oreganos in normal rats, conducted *in vivo*

The ex vivo oral glucose tolerance test (OGTT) was performed on healthy rats to evaluate the extract's ability to reduce increased blood glucose levels after a high glucose challenge. Rats with a weight ranging from 200 to 250 g were subjected to a 14-hour fasting period. They were then separated into four groups, each consisting of 6 rats with an equal number of males and females. The Control group was administered 10 mL/kg of distilled water, the Extract group was given 2 mL/kg of an aqueous extract, and the Glib group got 2 mg/kg of glibenclamide. At first, rats were rendered unconscious via ether inhalation, and their initial blood glucose levels were measured from the tail at t_0_. Afterward, the specific test chemicals were given by mouth. After a period of 30 min, a high dosage of D-glucose (2 mg/kg) was administered by injection, and blood glucose levels were observed every 30 min for a total of 150 min, which corresponds to a length of 3 h.

### 2.13 Statistical analysis

The results were reported as the average value ± the standard error of the average. The statistical analysis consisted of doing a one-way analysis of variance (ANOVA) followed by Tukey's post hoc test. This study was completed using GraphPad Prism 9 software (version 9.5.1) developed by GraphPad Software Inc. in San Diego, CA, USA. The significance level was established as *p < 0.05*.

## 3 Results

### 3.1 Phytochemical screening

Phytochemical analyses have revealed that the flowering tops of the investigated *Origanum* species include notable secondary metabolites (Table 2). The existence of sterols, triterpenes, flavonoids, gallic tannins, catechic tannins, and anthracene derivatives was confirmed by a highly favorable response. In addition, there was an adverse response seen for saponins and alkaloids.

**Table 2:** Results of phytochemical tests on the flowering tops of the studied oregano species.

**Compounds/species**		***O. compactum***	***O. elongatum***
**Sterols and triterpenes**		+++	++
**Flavonoids**		+++	+++
**Tannins**	Catechic tannins	++	+
	Gallic tannins	+	++
**Anthracene derivatives**	Quinones	+	+
	O-Heterosides	+	+
	C-Heterosides	++	++
**Saponosides**		−	−
**Alkaloids**	Dragendorff	−	−
	Mayer	−	−

Classification: A strong presence is shown by +++, a moderate presence by ++, a low presence by +, and an absence by −.

### 3.2 Phenolic compounds extraction and quantitative analysis

#### 3.2.1 Extraction yields

**Fig. 2:**
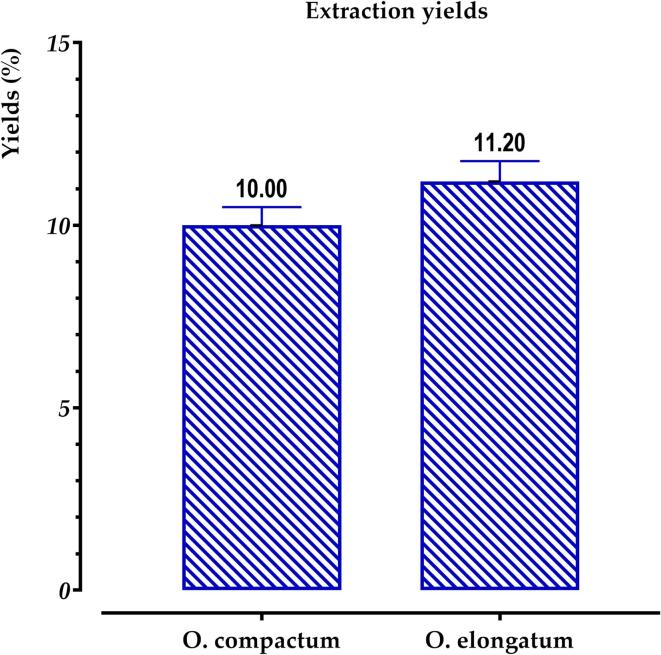
Displays the extraction yields of phenolic compounds from *O. compactum* and *O. elongatum.*

A solid–liquid extraction via decoction was conducted to evaluate the yields and concentrations of phenolic components between the two species. Based on Fig. 2, it seems that the yield of extracts obtained by decoction from the flowering tops of *O. elongatum* is somewhat greater than those obtained for *O. compactum*.

#### 3.2.2 Determination of phenolic compounds

Calibration curves were established to quantify the concentrations of polyphenols, flavonoids, condensed tannins, and hydrolyzable tannins in the aqueous extracts of the examined species, utilizing gallic acid (Y = 0.095X + 0.003; R^2^ = 0.998), quercetin (Y = 0.073X − 0.081; R^2^ = 0.995), catechin (Y = 0.7421X + 0.0318; R^2^ = 0.998), and tannic acid (Y = 0.1700X − 0.0006718; R^2^ = 0.996). The quantities of total polyphenols, flavonoids, condensed tannins, and hydrolyzable tannins in the extracts were quantified in milligrams per gram of extract, using gallic acid, quercetin, vanillin, and tannic acid equivalents as reference standards, respectively.

**Fig. 3:**
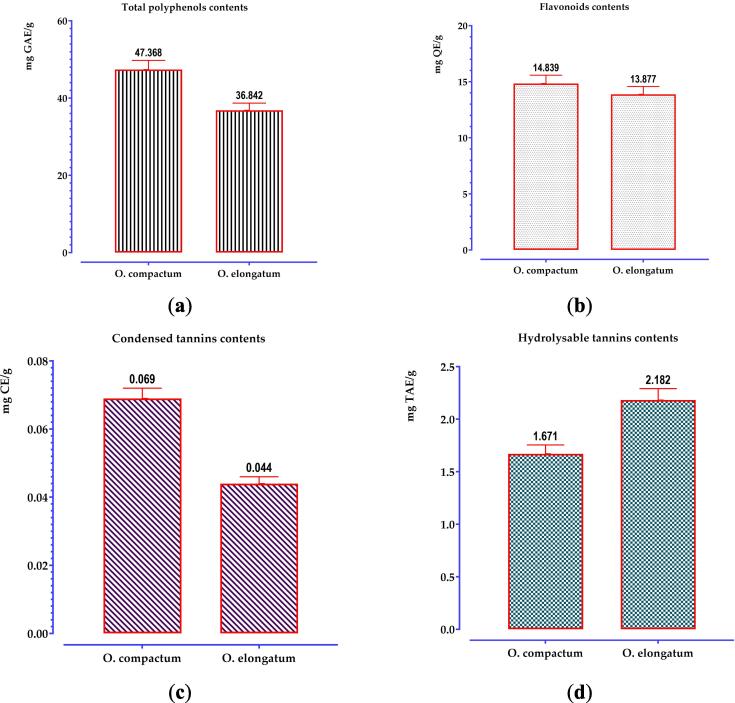
Quantities of phenolic compounds in the oregano decoctions examined: (a) total polyphenols; (b) flavonoids; (c) condensed tannins; (d) hydrolyzable tannins. The mean values together with their corresponding standard deviations, obtained from three separate determinations, are provided. The means display a significant difference (*p < 0.001*).

The overall polyphenol analysis findings indicate a notable disparity in amounts across the extracts (Fig. 3). The extract of *O. compactum* had the maximum concentration, reaching 47.368 mg GAE/g, but the decocted extract of *O. elongatum* showed somewhat lower levels.

Regarding the flavonoid contents (Fig. 3), both oregano extracts are significantly abundant in flavonoids, with the extract of *O. compactum* having the greatest quantities at 14.839 mg QE/g.

Analysis of tannin results indicates that the flowering tops are less rich in tannins (both condensed and hydrolyzable). The extracts obtained from *O. compactum* show the highest concentration of condensed tannins (0.069 mg EC/g of extracts), while the decocted extract of *O. elongatum* exhibits a higher content of hydrolyzable tannins (2.182 mg TAE/g of extracts).

#### 3.2.3 Analysis and identification of polyphenols in the decoctions of the studied oregano species by high-performance liquid chromatography coupled with mass spectrometry (HPLC/UV-ESI-MS)

**Fig. 4:**
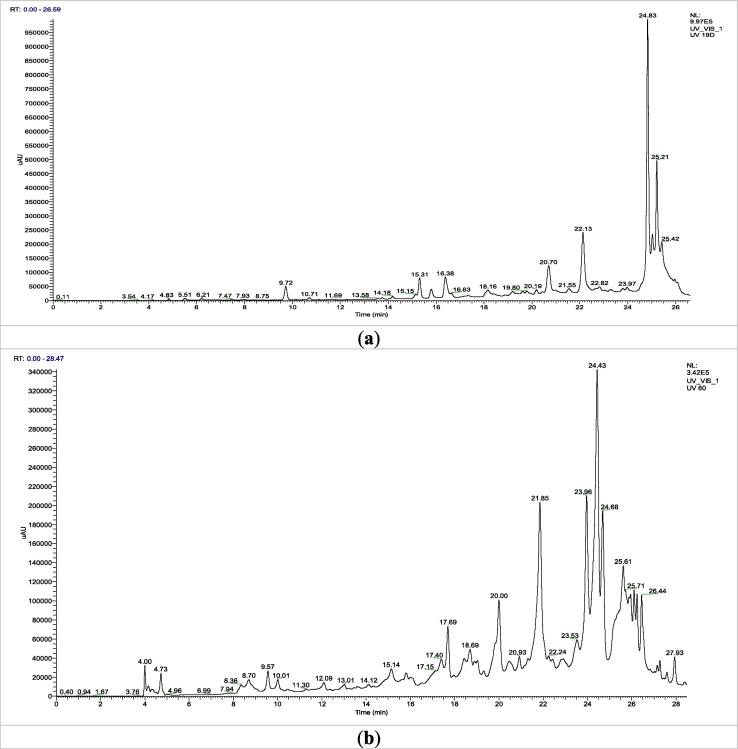
HPLC chromatograms of aqueous extract *Origanum* compounds: (a) *O. compactum*; (b) *O. elongatum.*

**Fig. 5:**
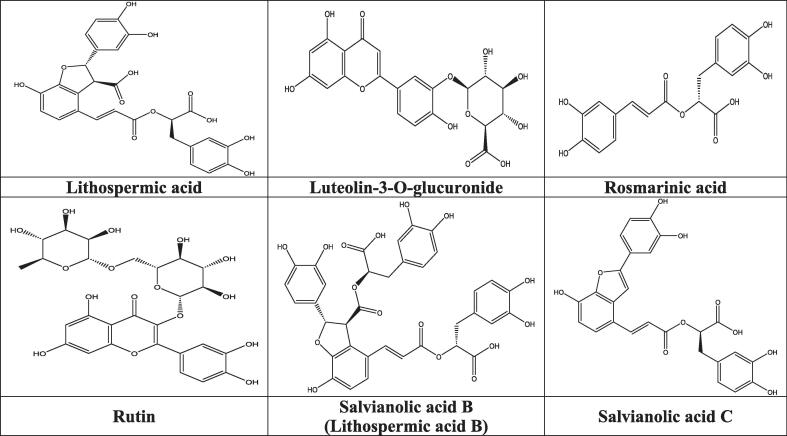
Structure of the main compounds identified in the aqueous extracts of the flowering tops of the analyzed *Origanum* species.

The HPLC/UV-ESI-MS technique was used to evaluate the aqueous extracts of the oregano species under study. The Fig. 4 shows the chromatogram, which reveals the various chemicals identified in the flowering tops of *O. compactum* and *O. elongatum*. By analyzing the mass spectra alongside the chromatogram, a total of 41 molecules were discovered, and are listed in Table 3. The primary chemicals that have been discovered are displayed in Fig. 5.

**Table 3:** List of Compounds Identified in Aqueous Extracts of Flowering Tops of Studied Oregano Species by HPLC/UV-ESI-MS in Negative Mode.

N°	RT (min)	Molecules	Classes	[M−H]- (*m*/*z*)	MS [M−H]- *m*/*z*	Area (%)	
						*O. compactum*	*O. elongatum*
1	4	Quinic acid	Organic acid	191	177-159-129	1,14	0,52
2	4,73	Citric acid	Organic acid	191	191-147-111	1,45	1,11
3	9,57	caffeic acid	Phenolic	179	135-107	0	1,46
4	9,72	Syringic acid	Phenolic	197	197-179-135	1,20	1,06
5	15,31	Gallocatechin	Flavonoid	305	305-225-191	1,99	2,23
6	15,81	1-Hexanol-pentosylhexoside	Terpenes	395	350-230-202	0	1,87
7	16,01	3-O-Caffeoylquinic acid	Phenolic	353	353-191-179-173-135	0	0,80
8	16,38	Methyl rosmarinate	Phenolic	373	373-197 - 179-135	3,61	1,75
9	17,35	Thymoquinol-O-hexoside	Phenolic	327	327-165 - 149-101	0,86	0
10	17,4	Luteolin-7-O-rutinoside	Flavonoid	593	593-285	0	3,17
11	17,69	Medioresinol	Lignans	387	387-305-207-179	1,44	2,06
12	18,16	luteolin-7-O-glucoside	Flavonoid	447	447-329 - 285	1,82	0
13	18,4	Luteolin 7,3′-di-O-glucuronide	Flavonoid	637	351-285-193-175	1,33	0
14	18,43	Rosmarinic acid sinapoyl-hexoside	Phenolic	727	529-359	0	2,63
15	18,69	3-O-Feruloylquinic acid	Phenolic	367	367-193	0	0,71
16	19,2	Tuberonic acid	Fatty acid	225	225-147	1,34	1,06
17	19,31	3′,4′,5′,5,7-Pentamethoxyflavone	Flavonoid	371	371-341	0	0,74
18	19,61	Eriodictyol-7-O-glucoside	Flavonoid	449	449-377-287-153	1,03	0
19	19,8	Sagerinic acid	Phenolic	719	539-521- 359-197- 179	1,54	1,21
20	20	Quercetin-3-O-glucoside	Flavonoid	463	463-301	0	5,60
21	20,19	Rosmarinic acid hexoside	Phenolic	521	521-359-197-179- 162)	1,28	0
22	20,44	Apigenin	Flavonoid	269	269-225-151-119	0,90	0
23	20,47	(+)-Catechin hydrated	Flavonoid	307	307-227-96	0	2,15
24	20,7	Salvianolic acid F isomer	Phenolic	313	269-203-161	4,41	0
25	20,93	3-O-methyl-catechin	Flavonoid	303	303-96	0	1,53
26	20,98	Salvianolic acid C derivative	Phenolic	715	715-491- 311-179	1,70	0
27	21,55	Salvianolic acid B isomer (I)	Phenolic	717	519-321	2,43	0
28	21,85	Luteolin-3-O-glucuronide	Flavonoid	461	461-387-285	0	13,51
29	22,81	Salvianolic acid C	Phenolic	491	311-135	12,57	14,46
30	22,91	Maslinic acid	Terpenes	471	471-451-407	0	1,18
31	23,53	Salvianolic acid K	Phenolic	555	537-493- 449-359- 313-269	0	1,52
32	23,96	Caffeic acide derivative	Phenolic	415	415-371-179	0	3,39
33	24,43	Rosmarinic acid	Phenolic	359	359-197-179-161-135	6,01	7,83
34	24,43	Retusin	Flavonoid	357	357-327-297	0	1,42
35	24,68	Rutin	Flavonoid	609	463-301	0	6,18
36	24,83	Lithospermic acid	Phenolic	537	537-493- 356-295	36,82	0
37	25,21	Salvianolic acid B isomer (II)	Phenolic	717	519-359-339	9,91	0
38	25,42	Dehydrocarnosol	Diterpene	327	327-299-284-269	1,40	2,28
39	25,61	Salvianolic acid B	Phenolic	717	717-519-421-339-321-179	0	12,24
40	25,97	Salvianolic acid A	Phenolic	493	493-313-295-185	2,48	2,08
41	26,07	Kaempferol 3-glucuronide	Flavonoid	461	461-285-229-113	0,97	1,97

The in-depth analysis of the mass spectra (HPLC/UV-ESI-MS) of the aqueous extracts of *O. compactum* and *O. elongatum*, performed in negative ionization mode, highlights the presence of several phenolic compounds and flavonoids.

For *O. compactum*, the main compounds identified are lithospermic acid (36.82 %), salvianolic acid C (12.57 %), an isomer of salvianolic acid B (9.91 %), and rosmarinic acid (6.01 %). Regarding *O. elongatum*, the composition is dominated by salvianolic acid C (14.46 %), luteolin-3-O-glucuronide (13.51 %), salvianolic acid B (12.24 %), rosmarinic acid (7.83 %), and rutin (6.18 %).

Furthermore, the presence of terpenes, fatty acids, and other organic acids was also observed in both species, although their percentages are relatively low. This study highlights the richness and diversity of bioactive compounds present in these two *Origanum* species, emphasizing their potential for various pharmacological and therapeutic applications.

Mass spectrometry analysis using electrospray ionization in negative ion mode (ESI-MS) is a crucial method for elucidating the structure of various organic molecules. This technique allows for the observation of the fragmentation of these compounds under ionization, thereby providing valuable information on their molecular architecture.

Regarding lithospermic acid, the ESI-MS spectrum revealed that this molecule undergoes ionization resulting in the loss of a proton (H-), forming the molecular ion [M−H]- observable at *m*/*z* = 537 and eluted at 24.83 min. The main fragments identified indicate a progressive fragmentation of this complex structure, beginning with the loss of a carboxyl group (–COOH), representing a mass decrease of 44 u, leading to the fragment at *m*/*z* 493. This initial step is followed by the cleavage of the bond between the aromatic rings, with the concomitant loss of an 181 u fragment, generating the fragment at *m*/*z* 356. The fragmentation continues with the elimination of a new 61 u fragment, resulting in an ion at *m*/*z* 295.

For luteolin-3-O-glucuronide, eluted at 21.85 min, the [M−H]- ion is detected at *m*/*z* 461. The main fragments first show the loss of the glucuronide group, representing a mass decrease of 174 u, leading to the fragment at *m*/*z* 387. This step is followed by the cleavage of the characteristic C-ring of the flavonoid structure, yielding the fragment at *m*/*z* 285.

Rosmarinic acid, eluted at 24.43 min, presents a molecular ion [M−H]- observed at *m*/*z* 359. The main identified fragments correspond to the progressive loss of the caffeic fragment, representing 162 u, leading to the fragment at *m*/*z* 197. This fragment then undergoes the successive loss of two water molecules, generating fragments at *m*/*z* 179 and 161. Further cleavage of the aromatic ring finally leads to the fragment at *m*/*z* 135.

Regarding rutin, eluted at 24.68 min, the [M−H]- ion is observed at *m*/*z* 609. The main fragments result from the loss of the rhamnose group, which is 146 u, giving the fragment at *m*/*z* 463, followed by the loss of the glucose group, representing 162 u, generating the characteristic quercetin fragment at *m*/*z* 301.

The molecular ion [M−H]- of salvianolic acid B, eluted at 25.61 min, is detected at *m*/*z* 717. Its fragmentation results in the successive loss of two caffeic groups, 198 u and then 98 u, leading to fragments at *m*/*z* 519 and 421. The skeleton then undergoes further cleavage, giving the fragment at *m*/*z* 339, which loses a water molecule to produce the fragment at *m*/*z* 321. Finally, the characteristic fragment of caffeic acid is observed at *m*/*z* 179.

The isomer of salvianolic acid B, eluted at 25.21 min, also presents a [M−H]- ion at *m*/*z* 717. Its main fragments are similar, with the loss of a caffeic group of 198 u resulting in the fragment at *m*/*z* 519, followed by further cleavage of the skeleton leading to the fragment at *m*/*z* 359. This fragment then loses a CO2 group of 44 u to give the fragment at *m*/*z* 339.

Finally, the [M−H]- ion of salvianolic acid C, eluted at 22.81 min, is observed at *m*/*z* 491. Its main fragments result from the loss of a water molecule (18 u) and a caffeic group (162 u), giving the fragment at *m*/*z* 311, as well as the characteristic fragment of caffeic acid at *m*/*z* 135.

### 3.3 Heavy metal contents

To date, little study has concentrated on the examination of heavy metal concentrations in the *Origanum* genus. Our research analyzed seven particular elements: arsenic (As), iron (Fe), cadmium (Cd), antimony (Sb), chromium (Cr), lead (Pb), and titanium (Ti). The concentrations of these components in the two samples were within the permitted limits set by the FAO/WHO (Table 4).

**Table 4:** Concentrations of heavy metals (mg/L) and maximum permissible limits according to FAO/WHO (2009).

**Species**	**Arsenic (As)**	**Cadmium (Cd)**	**Chromium (Cr)**	**Iron (Fe)**	**Lead (Pb)**	**Antimony (Sb)**	**Titanium (Ti)**
*O. compactum*	0,007	0,005	0,047	12,83	0,971	0,019	0,068
*O. elongatum*	0,1647	0,0452	0,0723	2,094	0,103	0,125	0,099
*Maximum limits (FAO/WHO)*	1	0.3	2	20	3	1	−

### 3.4 Antioxidant activity

The antioxidant properties of aqueous extracts from the oregano species examined were evaluated against ascorbic acid as a reference, using three distinct methods: DPPH, FRAP, and TAC. Calibration curves for ascorbic acid were established by the DPPH assay (Y = 1.013X − 8.032; R^2^ = 0.9893), the FRAP technique (Y = 0.004760X + 0.09740; R^2^ = 0.8963), and the TAC method (Y = 0.04066X + 0.02110; R^2^ = 0.9949).

**Fig. 6:**
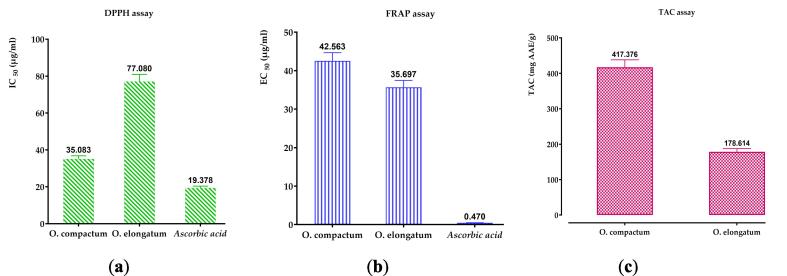
Displays the antioxidant activity of ascorbic acid and extracts using three different assays: (a) DPPH, (b) FRAP, and (c) TAC. The mean results, together with their corresponding standard deviations, are provided based on triplicate measurements. The means exhibit a substantial difference (*p < 0.001*).

These extracts are being investigated for their potential as natural antioxidants because of their capacity to diminish and/or prevent the generation of free radicals. The results shown in Fig. 6A indicate that the aqueous extracts of both *Origanum* species have strong antiradical action. The aqueous extract of *O. compactum* showed significant antioxidant activity, with an IC_50_ value of 35.083 μg/mL, whereas the extract of *O. elongatum* had an IC_50_ value of 77.080 μg/mL. As a point of comparison, the IC_50_ value of ascorbic acid is 19.378 μg/mL. In contrast, the findings obtained from the FRAP approach, as shown in Fig. 6B, demonstrate notable distinctions in the reducing capacity of the examined extracts compared to the positive control (ascorbic acid). Therefore, the aqueous extract of *O. elongatum* demonstrated a greater capacity for reducing power (35.697 μg/mL) in comparison to *O. compactum* (42.563 μg/mL), but these values are still lower than those of ascorbic acid (0.470 g/mL). Fig. 6C demonstrates the overall antioxidant capacity (TAC) of the extracts, quantified in terms of ascorbic acid equivalents. The extract of *O. compactum* exhibited a significantly greater total antioxidant capacity compared to the extract of *O. elongatum*. The results highlight the exceptional antioxidant capacity of the aqueous extracts from both oregano species, with the *O. compactum* extract demonstrating superiority in the majority of the conducted tests.

### 3.5 Antimicrobial activity

The findings of the antibacterial activity of the examined decoctions are shown in Table 5. The Minimum Inhibitory Concentration (MIC) of the extracts was assessed according to the standards set out by Sartoratto, Duarte, Wang, Oliveira, and their associates' colleagues (de Oliveira Pedro et al., 2013, Duarte et al., 2007, Sartoratto et al., 2004, Wang et al., 2017). Antimicrobial activity was classified as high (MIC < 600 μg/mL), moderate (MIC between 600 and 2500 μg/mL), or low (MIC > 2500 μg/mL).

**Table 5:** Minimum Inhibitory Concentration (MIC), Minimum Bactericidal Concentration (MBC), and Minimum Fungicidal Concentration (MFC) (µg/mL) of the decoctions from the examined oregano species, alongside the MIC values for antibiotics and antifungal agents.

***Microorganism***		***O. elongatum***		***O. compactum***		Antibiotics *					***Antifungals***#
		***MIC***	***MBC or MFC***	***MIC***	***MBC or MFC***	***Gentamycin***	***Amoxicillin–Clavulanate***	***Vancomycin***	***Trimethoprim-Sulfamethoxazole***	***Penicillin G***	***Terbinafine***
***GPC***	***S. epidermidis***	2500	2500	2500	5000	2		>8	>4/76		
	***S. aureus BLACT***	2500	2500	1200	1200	<0.5		2	<10		
	***S. aureus STAIML/MRS/mecA/HLMUP/BLACT***	>5000	>5000	5000	5000	2		>8	>4/76		
	***S. acidominimus***	300	600	75	150	≤250		<0.5		0.03	
	***S. group D***	600	600	600	600	>1000		<0.5		0.13	
	***S. agalactiae (B)***	1200	2500	600	1200	≤250		>4		0.06	
	***S. porcinus***	2500	2500	5000	5000	≤250		<0.5		0.06	
	***E. faecalis***	600	1200	300	300	≤500		1	≤0.5/9.5		
	***E. faecium***	5000	5000	5000	5000	≤500		>4	>4/76		
***GNB***	***A. baumannii***	300	300	150	300	≤1	≤2/2		≤1/19		
	***E. coli***	1200	1200	300	600	2	8/2		≤1/19		
	***E. coli ESBL***	1200	1200	600	600	2	>8/2		>4/76		
	***E. aerogenes***	1200	2500	1200	1200	≤1	8/2		≤1/19		
	***E. cloacae***	75	150	150	300	>4	>8/2		>4/76		
	***C. koseri***	75	150	150	150	<1	>8/2		<20		
	***K. pneumoniae***	600	1200	600	600	≤1	≤2/2		≤1/19		
	***P. mirabilis***	2500	5000	1200	2500	2	≤2/2		>1/19		
	***P. aeruginosa***	300	300	75	150	2	>8/2		4/76		
	***P. fluorescence***	1200	2500	600	600	4	>8/2		4/76		
	***P. putida***	2500	5000	600	1200	>4	>8/2		>4/76		
	***S. marcescences***	1200	2500	2500	5000	4	>8/2		>4/76		
	***Salmonella*** sp.	1200	2500	150	300	>4	8/2		>4/76		
	***Shigella*** sp.	600	1200	300	600	>4	8/2		>4/76		
	***Y. enterolitica***	300	300	300	600	≤1	8/2		2/38		
***Yeasts***	***C. albicans***	600	600	600	600						12.500
	***C. kefyr***	2500	2500	1200	1200						25.000
	***C. krusei***	5000	5000	2500	5000						50.000
	***C. parapsilosis***	1200	1200	600	1200						6.250
	***C. tropicalis***	1200	1200	600	600						12.500
	***C. dubliniensis***	1200	1200	150	300						3.125
	***S. cerevisiae***	2500	2500	2500	2500						3.125
***Molds***	***A. niger***	150	300	75	150						3.125

*The minimum inhibitory concentration (MIC) of the antibiotics was evaluated using the BD Phoenix™ identification and antibiogram device;

# The MIC of terbinafine was calculated using a microplate.

The analysis of the antimicrobial activity of extracts from *O. elongatum* and *O. compactum* reveals significant differences depending on the tested microorganisms. For Gram-positive cocci, *O. elongatum* proves more effective against Staphylococcus epidermidis, whereas *O. compactum* shows better performance against beta-lactam-resistant *Staphylococcus aureus* and *Streptococcus acidominimus*. However, both extracts are less effective against highly resistant strains of S. aureus.

In Gram-negative bacilli (GNB), the extract of *O. compactum* generally exhibits better antimicrobial activity compared to *O. elongatum* against most tested GNB. Specifically, against *Acinetobacter baumannii*, the MIC of *O. compactum* is lower at 150 μg/mL compared to 300 μg/mL for *O. elongatum*, indicating greater efficacy. Similarly, for *Escherichia coli*, the MIC and MBC values of *O. compactum*, 300 μg/mL and 600 μg/mL respectively, are significantly lower than those of *O. elongatum*, which are 1200 μg/mL for both parameters. However, both extracts showed comparable activity against ESBL-producing *Escherichia coli*, with similar MIC and MBC values. For *Enterobacter aerogenes*, both extracts also showed equivalent results, with identical MIC and MBC values of 1200 μg/mL. Conversely, *O. elongatum* was found more effective than *O. compactum* against Enterobacter cloacae. For most other tested GNBs, such as *Proteus mirabilis, Pseudomonas aeruginosa,* and *Citrobacter koseri*, *O. compactum* demonstrated notable superiority.

For yeasts, both extracts showed good efficacy against *Candida albicans*, with a MIC of 600 μg/mL. However, against *Candida krusei*, *O. compactum* demonstrated less effectiveness, with a MIC of 2500 μg/mL, compared to 5000 μg/mL for *O. elongatum*. Lastly, against the mold *Aspergillus niger*, *O. compactum* again exhibited superiority, with a MIC of 75 μg/mL, compared to 150 μg/mL for *O. elongatum*.

### 3.6 Anticoagulant activity

**Fig. 7:**
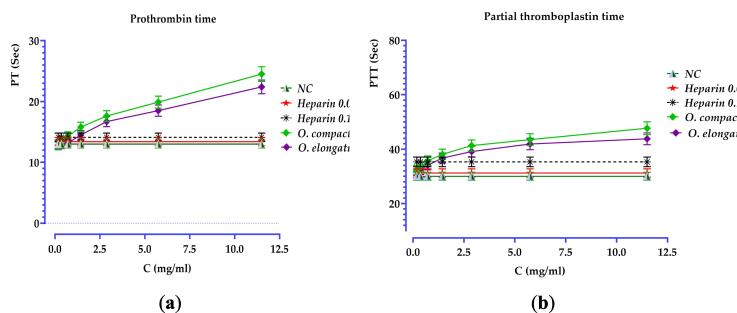
Impact of extracts, normal control (NC), and heparin on prothrombin time (a) and partial thromboplastin time (b).

According to the results shown in Fig. 7, it is clear that the prothrombin time (PT) increased in a manner that was dependent on the dosage for both the species *O. compactum* and *O. elongatum*. The PT test showed a significant increase in coagulation time with *O. compactum* compared to *O. elongatum*. The *O. compactum* had a PT value of 24.5 ± 1.22 s at a concentration of 11.5 mg/mL, whereas *O. elongatum* had a PT value of 22.4 ± 1.12 s. In addition, the aPTT test findings for the aqueous extracts showed significant anticoagulant action via blocking the intrinsic route. All extracts have shown a substantial ability to extend the activated partial thromboplastin time (p < 0.001) when the concentration reached 1.438 mg/mL.

**Fig. 8:**
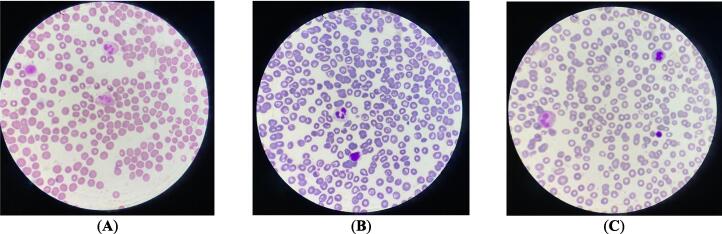
Blood Smears (100×): (A) Untreated control sample, (B) Sample treated with 11,500 mg/mL of *O. compactum* decoctate, (C) Sample treated with 11,500 mg/mL of *O. elongatum* decoctate.

The evaluation of blood parameters treated with *O. compactum* and *O. elongatum* decoctions at various concentrations (ranging from 0.179 mg/mL to 11.5 mg/mL) revealed a slight decrease in the number of leukocytes, red blood cells, hemoglobin, and platelets, particularly at the highest concentration of 11.5 mg/mL (Table 6 **and** Fig. 8). Specifically, the leukocyte count decreased from 6.5 ± 0.3 × 10^9^/L in the control sample to 5.9 ± 0.2 × 10^9^/L with *O. compactum* and to 5.7 ± 0.2 × 10^9^/L with *O. elongatum*. Similarly, the red blood cell count decreased from 4.37 ± 0.2 × 10^12^/L in the control to 3.99 ± 0.1 × 10^12^/L with *O. compactum* and to 3.96 ± 0.1 × 10^12^/L with *O. elongatum*. Hemoglobin and hematocrit levels also showed a slight reduction, respectively decreasing from 12.9 ± 0.6 g/dL to 11.9 ± 0.5 g/dL and from 38.4 ± 1.9 % to 36.4 ± 1.7 % with *O. compactum* and to 35.2 ± 1.7 % with *O. elongatum*. The platelet count decreased from 239 ± 12.0 × 10^9^/L in the control to 216 ± 10.8 × 10^9^/L with *O. compactum* and to 219 ± 12.4 × 10^9^/L with *O. elongatum*. However, the mean platelet volume and platelet dispersion index remained stable, indicating no notable changes. To summarize, the *Origanum* decoctions resulted in a little decrease in specific hematological parameters at elevated doses, while having no impact on the average platelet volume or the platelet dispersion index.

**Table 6:** Results of blood counts for samples studied.

Blood Count		*O. compactum*							*O. elongatum*						
		**Decocted Concentration (mg/mL)**													
**Parameters**	**Control Sample**	**0.179**	**0.359**	**0.719**	**1.438**	**2.875**	**5.75**	**11.5**	**0.179**	**0.359**	**0.719**	**1.438**	**2.875**	**5.75**	**11.5**
**Leukocytes**	6.5 ± 0.3	6,2 ± 0,3	6,1 ± 0,2	6,3 ± 0,3	6,3 ± 0,3	6,2 ± 0,3	6,1 ± 0,3	5,9 ± 0,2	6,2 ± 0,3	6,1 ± 0,3	6,3 ± 0,3	6,3 ± 0,3	6,2 ± 0,3	6,1 ± 0,3	5,7 ± 0,2
**Red blood cells**	4.37 ± 0.2	4,17 ± 0,2	4,19 ± 0,2	4,17 ± 0,2	4,19 ± 0,2	4,21 ± 0,2	4,2 ± 0,2	3,99 ± 0,1	4,16 ± 0,2	4,19 ± 0,2	4,17 ± 0,2	4,18 ± 0,2	4,18 ± 0,2	4,18 ± 0,2	3,96 ± 0,1
**Hemoglobin**	12.9 ± 0.6	12,2 ± 0,6	12,2 ± 0,5	12,3 ± 0,6	12,2 ± 0,6	12,3 ± 0,6	12,4 ± 0,6	11,9 ± 0,5	12,1 ± 0,6	12,2 ± 0,6	12,2 ± 0,6	12,2 ± 0,6	12,2 ± 0,6	12,2 ± 0,6	11,8 ± 0,5
**Hematocrit**	38.4 ± 1.9	37,5 ± 1,8	37,3 ± 1,9	36,9 ± 1,8	37,1 ± 1,8	37,2 ± 1,8	37,1 ± 1,8	36,4 ± 1,7	37,4 ± 1,8	37,0 ± 1,8	36,8 ± 1,8	37,0 ± 1,8	37,0 ± 1,8	36,9 ± 1,8	35,2 ± 1,7
**Platelets**	239 ± 12.0	226 ± 11,3	228 ± 11,2	231 ± 11,5	220 ± 11.3	223 ± 10,6	219 ± 10,9	216 ± 10,8	236 ± 13,3	228 ± 12,9	231 ± 13,0	229 ± 12,4	222 ± 13,6	225 ± 13,7	219 ± 12,4
**Mean platelet volume**	9.4 ± 0.5	9,2 ± 0,4	9,1 ± 0,4	9,2 ± 0,4	9,1 ± 0,4	9,2 ± 0,4	9,1 ± 0,4	9,3 ± 0,4	9,2 ± 0,4	9,0 ± 0,4	9,2 ± 0,4	9,1 ± 0,4	9,1 ± 0,4	9,0 ± 0,4	9,2 ± 0,4
**Platelet distribution index**	14.7 ± 0.7	14,6 ± 0,7	14,5 ± 0,7	14,7 ± 0,6	14,5 ± 0,6	14,7 ± 0,7	14,7 ± 0,7	14,6 ± 0,7	14,6 ± 0,7	14,5 ± 0,7	14,7 ± 0,7	14,4 ± 0,7	14,6 ± 0,7	14,6 ± 0,7	14,6 ± 0,7

### 3.7 Antidiabetic activity

#### 3.7.1 Evaluation of the *in vitro* inhibitory effects of decocted extracts on α-amylase and α-glucosidase activities

**Fig. 9:**
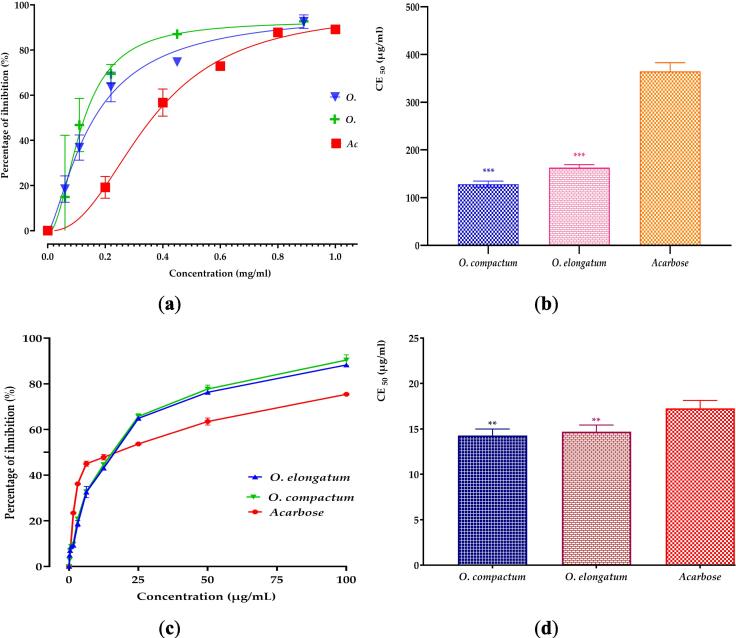
*In Vitro* Inhibitory Effects of Decoctions from Studied *Origanum* Species and Acarbose on α-Amylase (a,b) and α-Glucosidase (c,d) Activities: Percentage Inhibition and EC_50_ Values. Mean values ± SEM (n = 3).

The assessment of the inhibitory effects of extracts from *Origanum* species on α-amylase and α-glucosidase activities under controlled conditions demonstrated significant, dose-dependent inhibition, as illustrated in Fig. 9. The results indicate that the aqueous extracts exhibit substantial inhibitory effects on both enzymes, surpassing even the reference inhibitor, acarbose. The extract of *O. compactum* showed strong inhibitory activity against α-amylase, with EC_50_ values of 128.134 mg/mL. *O. elongatum* also showed significant outcomes, with EC_50_ values of 162.805 mg/mL. However, acarbose, used as a reference, showed a gradual rise in inhibitory activity as the concentration rose. However, it only reached an EC_50_ value of 364.446 µg/mL for this specific enzyme. The EC_50_ values for inhibiting α-glucosidase were 14.277 µg/mL and 14.6954 µg/mL for the extracts of *O. compactum* and *O. elongatum*, respectively. These findings demonstrate that the inhibitory activity of the mentioned substances is equivalent and may be compared to that of acarbose, which exhibited an EC_50_ value of 17.269 µg/mL.

#### 3.7.2 Evaluation of the acute toxicity of decoctions made from *O. compactum* and *O. elongatum*

The results obtained from the acute toxicity investigation of decoctions from both species of oregano are highly comforting in this respect. These extracts have been proven to be non-toxic, even at large dosages of up to 2 g/kg. Throughout the monitoring period, there were no indications of toxicity, such as diarrhea, vomiting, or mobility impairments, identified in the individuals. Additionally, no deaths were documented among those who received these extracts. These findings are especially promising since they suggest that extracts from the oregano species being examined have a very positive safety profile. The extracts demonstrate a total lack of acute toxicity, even at higher dosages, indicating that they might be explored for potential therapeutic applications without significant worries about severe negative consequences. This further strengthens the enthusiasm for the interesting biological characteristics of these plants.

#### 3.7.3 Investigation of the antihyperglycemic effects of the examined decoctions in normal rats in a live setting

**Fig. 10:**
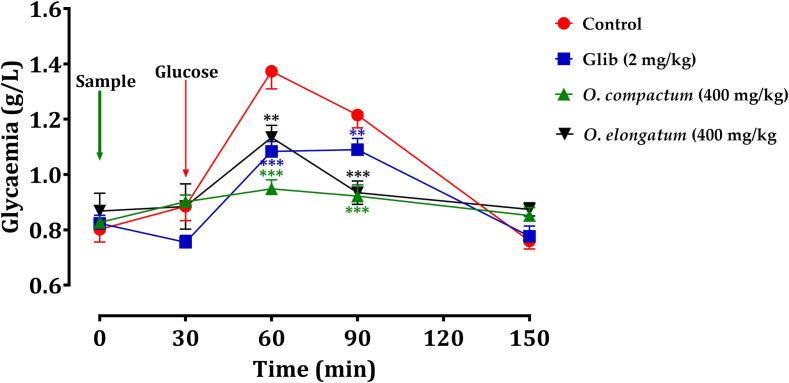
Variation in blood glucose levels after giving normal rats the test items (Extracts and glibenclamide). The values are the means with the standard error of the mean (SEM). The value of n is 6. ^**^p < 0.01, and ^***^p < 0.001 compared to the control group.

**Fig. 11:**
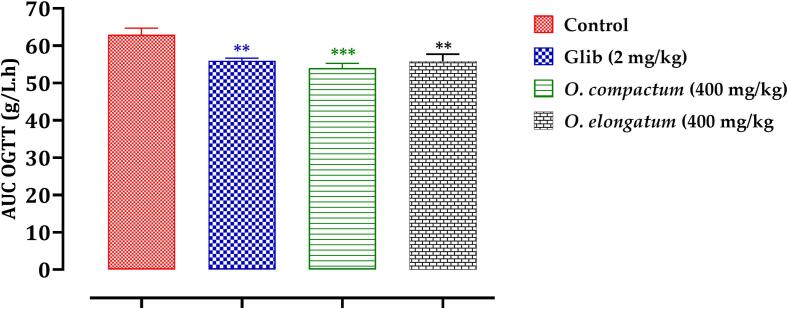
Variation in Postprandial Blood Glucose Area Under the Curve in Normal Rats Following Administration of Tested Products (Extracts and Glibenclamide). Mean values ± SEM. (n = 6). *p < 0.05 compared to control.

Research was undertaken to investigate the antihyperglycemic activity of the decoctions of *O. compactum* and *O. elongatum* in normal rats. This was done by performing an oral glucose tolerance test and comparing the area under the glucose curve (AUC) for 150 min. The results of this study are shown in Fig. 10, Fig. 11.

• Oral glucose tolerance test

The results indicate that in healthy rats, blood glucose concentrations reached their zenith 30 min after a glucose challenge. Nevertheless, animals who received the extracts plus glibenclamide exhibited notable antihyperglycemic benefits in comparison to control rats that were given distilled water beforehand. Administering orally ingested extracts at a dosage of 400 mg/kg, 30 min before the glucose overload, successfully alleviated postprandial hyperglycemia (Fig. 10). On the other hand, glibenclamide effectively suppressed the increase in blood sugar levels after a meal within the first hour (60 min) after consuming glucose, resulting in a substantial decrease in blood glucose levels (*p < 0.001*; 1.08 g/L).

• Postprandial glucose levels' areas under the curve (AUCs)

An examination of the area under the curve (AUC) showed that rats administered the extracts had a markedly reduced AUC (*p < 0.001*) in contrast to those receiving distilled water (62.91 g/L/h). The AUC for the glibenclamide-treated group was significantly decreased (55.95 g/L/h; p < 0.01) in comparison to the control rats (62.91 g/L/h) (Fig. 11). The postprandial blood glucose readings (in g/L/h) for the different groups were as follows: Control (62.91 g/L/h), glibenclamide (2 mg/kg; 55.95 g/L/h), *O. compactum* (400 mg/kg; 53.98 g/L/h), and *O. elongatum* (400 mg/kg; 55.82 g/L/h). The results suggest that the decocted extracts of the studied *Origanum* species have a notable antihyperglycemic effect, similar to glibenclamide, a commonly used antidiabetic medication. The authors emphasize the therapeutic potential of these extracts in controlling high blood sugar levels, indicating that they might be used as natural alternatives to traditional pharmaceutical therapies.

## 4 Discussion

The abundance of secondary metabolites, primarily sterols, triterpenes, flavonoids, and tannins, in the flowering tops of the *Origanum* species under study has been brought to light by phytochemical screening. These substances probably provide these plants with fascinating biological and medicinal qualities. These results are in line with previous research on the chemical makeup of the *Origanum* genus (Abdelaali et al., 2021, Bouyahya et al., 2020, Sharifi-Rad et al., 2021). Significant amounts of lipid molecules, including steroids and triterpenes, are present in both species. These compounds are important for the formation of cellular membranes and may potentially have positive biological effects, such as antioxidant qualities (Gonzalez-Burgos and Gomez-Serranillos, 2012, Summons et al., 2006). Both species contain large amounts of flavonoids, which are members of the polyphenol class and are known for their antibacterial, antidiabetic, and antioxidant qualities (Gutiérrez-Grijalva et al., 2018). Furthermore, differences in the tannin content may have an impact on the biological characteristics of both *Origanum* species. Tannins, whether of the gallate or catechol kind, are recognized for their antibacterial, antidiabetic, and antioxidant properties. Therefore, variations in the biological effects of *O. elongatum* and *O. compactum* may result from their different secondary metabolite profiles.

An analysis of phenolic components in decoctions of *O. compactum* and *O. elongatum* demonstrates significant variations between these two species, emphasizing the abundant and varied phenolic profiles present in the *Origanum* genus. The *O. compactum* is recognized for its remarkably elevated concentration of total polyphenols, measuring 47.368 mg gallic acid equivalents (GAE) per gram of extract. The measures of this species surpass those of other species in the same genus, such as *O. majorana*, *O. onites*, *O. hypericifolium*, and *O. sipyleum*, which range from 3.81 to 47.54 mg GAE/g (Semiz et al., 2018). In addition, previous research has documented elevated levels of concentration for methanolic extracts of *O. compactum*, precisely measured at 153.27 ± 0.68 mg GAE/g extract (Abdelhakim Bouyahya et al., 2017a). When compared, the results for *O. elongatum* are lower than the findings published by Douhri et al. (Badia et al., 2014), who achieved a value of 83.61 ± 0.19 mg GAE/g for the methanolic extract. *O. compactum* has a flavonoid content of 14.839 mg quercetin equivalents (QE) per gram of extract (decoction), which is greater than the published value of 9.04 mg QE/g for *O. majorana* according to Mahmah et al. (Aya et al., 2022). Nevertheless, the findings for *O. elongatum* are inferior to the results documented by Douhri et al. (Badia et al., 2014) for the methanolic extract. In addition, El Babili et al. (El Babili et al., 2011) observed significantly greater levels of flavonoid content in the ethyl acetate fraction (54.7 ± 1.8 mg QE/g extract) of *O. compactum*. This emphasizes the influence of the extraction process and solvents on the concentration of flavonoids. Moreover, the examination of tannins uncovers intriguing distinctions between the two species: *O. compactum* has a greater concentration of condensed tannins, but *O. elongatum* is more abundant in hydrolyzable tannins. On the other hand, *O. vulgare* is mostly composed of hydrolyzable tannins (Eltigani et al., 2020). Different types of tannins can have distinct effects, especially in terms of their antioxidant properties and interactions with other biomolecules. The variations in phenolic compound levels can be ascribed to the varied solubility of these compounds in various solvents. Ethanol and ethyl acetate exhibit higher extraction efficiency for these substances, although water is better suited for use in traditional medicine and pharmacopeias (Barros et al., 2010, Giacometti et al., 2018, Kumar et al., 2023, Milevskaya et al., 2019, Sasidharan et al., 2010). Hence, the flourishing top sections of *Origanum* species serve as a captivating repository of phenolic compounds, showcasing varied profiles among various species. The *O. compactum* is notable for its exceptionally high levels of total polyphenols and flavonoids, whereas *O. elongatum* has a unique tannin composition, mostly consisting of hydrolyzable tannins.

The detailed phenolic content of aqueous extracts derived from the flowering tops of *O. compactum* and *O. elongatum* was discovered using high-performance liquid chromatography coupled with mass spectrometry (HPLC/UV-ESI-MS) analysis. Both species possess distinct and varied phenolic profiles, spanning a wide array of chemicals. The examination of *O. compactum* decoction revealed the presence of lithospermic acid, salvianolic acid C, an isomer of salvianolic acid B, and rosmarinic acid as the primary components. These substances are classified as phenolic acids and are generally acknowledged for their strong antioxidant characteristics. This confirms previous research showing high concentrations of these phenolic compounds in other *Origanum* species, such as *O. majorana* (Gomes et al., 2020). Chroho et al. (Chroho et al., 2022) stressed the presence of phenolic acids, especially rosmarinic acid and lithospermic acid, in their investigation of *O. compactum* aqueous extracts. The values obtained in our study were found to be comparable to the findings. Their work also confirmed the potential medical value of *O. compactum* by demonstrating its antioxidant and antibacterial capabilities. In a similar vein, Msaada et al. (Msaada et al., 2017) discovered substantial quantities of rosmarinic acid, lithospermic acid, and other phenolic acids in extracts of *O. compactum*. This is consistent with our research and highlights the antioxidant and antibacterial properties of these chemicals. Bouyahya et al. (Bouyahya et al., 2020) just completed a thorough examination that proved the presence of lithospermic acid and salvianolic acid C in *O. compactum*, thereby establishing their prevalence. The study also demonstrated the strong antioxidant properties of these acids and their potential use in treating inflammatory and viral diseases. The therapeutic benefits of plants like *O. majorana* (Ayari et al., 2013) and *O. vulgare* (Blank et al., 2019, Oniga et al., 2018) are confirmed by the similarities in their chemical composition and biological features with *O. compactum*. This suggests that phenolic acids play a significant role in these plants' healing abilities. Thorough bibliographic comparisons back up our results, proving that *O. compactum* is a great source of natural antioxidants that might be used in plant-based products.

Salvinolinic acid C (14.46 %), luteolin-3-O-glucuronide (13.51 %), salvianolic acid B (12.24 %), rosmarinic acid (7.83 %), and rutin (6.18 %) make up the majority of O. elongatum's molecules. Due to the presence of flavonoids such as luteolin and rutin, these species possess remarkable antioxidant and free radical scavenging capabilities (Gutiérrez-Grijalva et al., 2018). Aqueous extracts of O. elongatum include high amounts of salvianolic acids C and B, lu-teolin-3-O-glucuronide, and rutin, which have been shown to have antioxidant properties, according to research by Hari et al. (Hari et al., 2024) and Kan et al. (Kan et al., 2014). These extracts have antibacterial properties, as shown by the high amounts of rosmarinic acid and salvianolic acid C discovered by Lin et al. (Lin et al., 2003). Studies conducted in China by Yu et al. (Yu et al., 2021) and Chile by Parra et al. (Parra et al., 2021) found that species belonging to the same genus had high quantities of flavonoids, which showed that they have significant antioxidant and anti-diabetic properties. Taken together, our results show that different species of *Origanum* produce phenolic chemicals in different amounts and with different qualitative profiles in their flowering tops. These variations might lead to different biological functions and possible uses.

As far as we know, there are few studies on the antioxidant and antibacterial properties of aqueous extracts from *Origanum* species. The antioxidant activities of the decoctions of *O. compactum* and *O. elongatum* were assessed using three complementary methods: the DPPH test, the FRAP assay, and the total antioxidant capacity (TAC) assay. These methodologies enable the evaluation of many elements of antioxidant activity, including the ability to scavenge free radicals, the power to reduce, and the capacity to neutralize oxidizing species. The findings of our study emphasize the exceptional ability of *Origanum* extracts to counteract the harmful effects of free radicals, which aligns with prior research conducted on different species of *Origanum*. For instance, Kulišić et al. (Kulišić et al., 2007) provided evidence that extracts from *O. vulgare* also had potent antioxidant properties, thereby supporting the effectiveness of the *Origanum* genus as an antioxidant. Moreover, the extracts' ability to reduce is strongly linked to the existence of phenolic chemicals that may donate hydrogen atoms. Previous research, such as that of Wojdyło et al. (Wojdyło et al., 2007), established a direct correlation between the phenolic content and the antioxidant activity of plants, supporting our observations. The extract of *O. compactum* demonstrated a very high total antioxidant capacity, surpassing that of the *O. elongatum* extract. This difference could be explained by the more diverse phenolic composition and the presence of more potent compounds in the *O. compactum* extract, such as lithospermic acid. A similar study by Exarchou et al. (Exarchou et al., 2002) on various *Origanum* species also found that variations in phenolic composition significantly influenced the antioxidant activity of the extracts. Moreover, it is noteworthy that these results are not isolated. A comparative study by Aboelmaati et al. (Aboelmaati et al., 2012) on extracts of various aromatic herbs showed that plants rich in phenolic compounds, like *O. majorana*, exhibited high antioxidant activity, comparable to that observed in our study for *O. compactum* and *O. elongatum*. This study provides evidence that plants belonging to the *Origanum* genus are abundant and potent producers of antioxidant chemicals. In addition, the findings of Bouyahya et al. (Abdelhakim Bouyahya et al., 2017c) showed that the methanolic and ethanolic extracts of *O. compactum* had significant antioxidant activity in scavenging DPPH radicals. Karimi et al. (Karimi et al., 2015) found a strong association between the antioxidant activity of oregano and its total phenol concentration. Furthermore, Baranauskaite et al. (Baranauskaite et al., 2017) demonstrated that the level of antioxidant activity in extracts obtained from three distinct *Origanum* species (*O. onites* L., *O. vulgare* L., and *O. vulgare* spp. hirtum) is species-dependent, which may be attributed to differing quantities of rosmarinic acid. The FRAP experiment indicates that the ability of *O. elongatum* to reduce is probably because it contains hydroxyl groups in phenolic compounds, which may act as electron donors. Many previous studies have attributed the antioxidant activity of different *Origanum* species to the major compounds in their extracts, such as flavonoids and phenolic acids (Bouyahya et al., 2017d, El-Ghorab et al., 2022). Lagouri and Alexandri (Lagouri and Alexandri, 2011) found a positive correlation between antioxidant activities by DPPH and FRAP assays and the total polyphenol content of hexane, methanol, and infusion extracts of *O. dictamnus*. Moreover, Kaliora et al. (Kaliora et al., 2014) reported a correlation between radical-scavenging activity and the total polyphenol content of leaf and flower infusions of *O. dictamnus*. Finally, another widely studied oregano species for its antioxidant activity is *O. vulgare*, with high values attributed to its rosmarinic acid, apigenin, caffeic acid, and rutin content (de Torre et al., 2020, Oniga et al., 2018). The aqueous extracts of both *Origanum* species possess outstanding antioxidant qualities, with the *O. compactum* extract demonstrating superiority in most performed studies. This implies possible applications in the pharmaceutical, cosmetic, and food industries. In a study done by Viuda-Martos et al. (Viuda-Martos et al., 2010), the researchers examined the use of *Origanum* extracts in food formulations to enhance the stability and quality of products, highlighting their practical utility. The findings of our study are consistent with the information presented in previous research, verifying the notable antioxidant capacity of the water-based extracts derived from *O. compactum* and *O. elongatum*. These qualities render these fragrant plants intriguing contenders for many applications, consequently augmenting their increased worth and significance for study and industry.

The examination of the antibacterial properties of infusions derived from *O. compactum* and *O. elongatum* demonstrates notable variations in effectiveness depending on the specific microorganisms under investigation. These findings highlight the intricate nature of the interactions between the phytochemical substances found in these extracts and different diseases. Regarding Gram-positive bacteria, our research suggests that the extract of *O. compactum* is generally more potent than that of *O. elongatum* since it has lower minimum inhibitory concentration and minimum bactericidal concentration values for most of the strains we examined. The difference in effectiveness may be ascribed to a greater concentration of bioactive phenolic components in the *O. compactum* extract. However, the variations seen in various bacterial species indicate that the antimicrobial chemical profiles of the two *Origanum* species may change, and their effectiveness is also influenced by the individual characteristics of the microorganisms. The findings align with the studies done by Brdjanin et al. (Brdjanin et al., 2015) and Duque-Soto et al. (Duque-Soto et al., 2023), which demonstrated the effectiveness of *Origanum* extracts in combating various Gram-positive bacterial infections. The efficacy of this is attributed to phenolic compounds, including caffeic acid, rosmarinic acid, ferulic acid, luteolin, apigenin, carvacrol, and thymol.

The findings of this study indicate that the extract of *O. compactum* typically displays superior antibacterial activity compared to that of *O. elongatum* against the tested Gram-negative bacilli (GNB), thus confirming earlier research on the antimicrobial capabilities of *Origanum* extracts. As an example, while testing against *Acinetobacter baumannii*, the *O. compactum* extract has a minimum inhibitory concentration of 150 µg/mL, whereas the MIC for *O. elongatum* is 300 µg/mL. *Escherichia coli* has considerably lower MIC and minimum bactericidal concentration values when exposed to the *O. compactum* extract compared to *O. elongatum*. More precisely, the MIC for *O. compactum* is 300 µg/mL, while the minimum bactericidal concentration is 600 µg/mL. However, for *O. elongatum*, both the MIC and minimum bactericidal concentration are measured at 1200 µg/mL. The findings are consistent with the study done by Boutahiri et al. (Boutahiri et al., 2022), which highlighted the notable antibacterial effects of water-based extracts from *O. compactum* against drug-resistant organisms such as *Escherichia coli.* Furthermore, Bouyahya et al. (Abdelhakim Bouyahya et al., 2017b) discovered that aqueous extracts derived from *O. compactum* had significant inhibitory properties against several strains of Gram-negative bacteria, including *Escherichia coli* and *Pseudomonas aeruginosa*. The *O. compactum* stands out because it contains high concentrations of bioactive phenolic compounds, such as rosmarinic acid, caffeic acid, quercetin, and apigenin. These chemicals are well-acknowledged for their potent antibacterial properties (Takó et al., 2020, Vijayakumar and Raja, 2022). Furthermore, improving the cooperation between the various constituents of this extract might augment its antibacterial efficacy, as shown by Saci et al. (Saci et al., 2024) and Soltani et al. (Soltani et al., 2021) in their investigations on extracts derived from *O. vulgare*. Moreover, it is crucial to underscore the remarkable efficacy of *O. compactum* against Gram-negative bacteria, especially those that exhibit resistance to various medicines. This underscores its potential as a viable alternative or complementary therapeutic agent to conventional therapies. This is particularly significant given the increasing prevalence of antibiotic-resistant bacterial infections. These discoveries highlight the significance of ongoing research on extracts from *O. compactum* and other medicinal plants. The objective is to identify their active components and evaluate their effectiveness in treating antibiotic-resistant bacterial infections. Both extracts of *Origanum* demonstrated substantial antifungal activity against *Candida albicans*, with a minimum inhibitory concentration of 600 µg/mL. However, in the case of *Candida krusei*, *O. compactum* showed lower potency, with a minimum inhibitory concentration of 2500 µg/mL, while *O. elongatum* had a MIC of 5000 µg/mL. Bhat et al. (Bhat et al., 2018) observed the differences in antifungal susceptibility across several *Candida* species. The authors suggested that these differences may be explained by species-specific mechanisms of resistance in *Candida*. Concerning the mold *Aspergillus niger*, *O. compactum* has once again shown greater sensitivity, with a minimum inhibitory concentration of 75 µg/mL, in comparison to *O. elongatum* which has a MIC of 150 µg/mL. Multiple investigations, like the one performed by Kocić-Tanackov et al. (Kocić-Tanackov et al., 2012), have shown the antifungal activities of *Origanum* extracts. This research mainly focused on evaluating the efficacy of aqueous extracts derived from different species of *Origanum* against diverse species of *Aspergillus*. These findings are consistent with the work of Boutahiri et al. (Boutahiri et al., 2022), who showed that aqueous extracts of *O. compactum* exhibited strong inhibitory activity against various species of yeasts and molds, notably *Candida albicans* and *Aspergillus niger*. According to these authors, phenolic compounds such as rosmarinic acid, caffeic acid, quercetin, and apigenin, as well as carvacrol and thymol, present in high quantities in *O. compactum*, are responsible for this antifungal activity.

These results suggest that *Origanum* decoctions possess interesting antimicrobial properties, particularly against certain problematic bacterial and fungal strains. The extract of *O. compactum* appears to be generally more effective than that of *O. elongatum*, especially against Gram-negative bacilli. According to Radulovic et al. (Radulovic et al., 2013), the mechanisms of action of these extracts may include the disruption of microbial cell membranes and the inhibition of essential enzymes. Nevertheless, further studies are necessary to better understand these processes and to optimize the potential use of these extracts in agri-food and biomedical applications.

The extracts of both *Origanum* species increased the prothrombin time (PT) in a way that depended on the dosage. The extract of *O. compactum* demonstrated the most significant extension of PT, with a value of 24.5 ± 1.22 s at a concentration of 11.5 mg/mL. This result surpassed the extract of *O. elongatum*, which exhibited a PT of 22.4 ± 1.12 s. The extension of the prothrombin time (PT) indicates that the extrinsic coagulation pathway is being inhibited, most likely as a result of the interaction between chemicals found in the extracts and coagulation factors. In addition, the activated partial thromboplastin time (aPTT) coagulation assays demonstrated significant anticoagulant activity of both extracts, effectively suppressing the intrinsic coagulation pathway. Indeed, all extracts significantly prolonged the aPTT (*p < 0.001*) from a concentration of 1.438 mg/mL. This anticoagulant effect on the intrinsic pathway could be explained by the interaction of compounds such as lithospermic acid, salvianolic acid derivatives, rosmarinic acid, caffeic acid, quercetin, apigenin, and rutin with coagulation factors (Orgah et al., 2020, Patrignani et al., 2021, Sławińska et al., 2023, Weiskirchen, 2016). These results corroborate those reported by Amin Mohamed et al. (Amin Mohamed et al., 2016) and Pour et al. (Pour et al., 2017). Moreover, both extracts notably prolonged the activated partial thromboplastin time (aPTT), suggesting a marked inhibition of the intrinsic coagulation pathway. This phenomenon could be explained by the interaction of phenolic compounds present in these extracts with the essential factors of this pathway, as indicated in the studies of Amin Mohamed et al. (Amin Mohamed et al., 2016) and Pour et al. (Pour et al., 2017). Curiously, these extracts with anticoagulant properties did not have a significant influence on hematological indicators, indicating that there were no harmful consequences at the dosages that were evaluated. The anticoagulant activities of *Origanum* extracts, as found in investigations conducted by Sharifi-Rad et al. (Sharifi-Rad et al., 2021), indicate their potential as attractive candidates for the development of novel natural thromboprophylactic drugs. These findings provide opportunities for future studies focused on using these extracts to create preventative therapies for coagulation problems. This might provide a potentially safe and effective alternative to traditional therapy choices.

The medicinal effects of *Origanum* species in the management of diabetes have been shown by Lemhadri et al. (Lemhadri et al., 2004), Bejaoui et al. (Bejaoui et al., 2016), and Soliman et al. (Soliman et al., 2016). The research has shown the significant inhibitory capacity of decocted extracts on key enzymes involved in carbohydrate metabolism, including α-amylase and α-glucosidase. Our analysis revealed that the extract of *O. compactum* had a significant inhibitory effect on α-amylase, with an EC_50_ value of 128.134 mg/mL. The value exceeded that of acarbose, a benchmark medication. The *O. elongatum* demonstrated substantial efficacy against α-amylase, with an EC_50_ value of 162.805 mg/mL. Both extracts exhibited comparable efficacy to acarbose in inhibiting α-glucosidase, with EC_50_ values of 14.277 µg/mL and 14.6954 µg/mL, respectively. Moreover, research conducted by Badekova et al. (Badekova et al., 2023) and Shokrzadeh (Shokrzadeh et al., 2014) has conclusively shown that *Origanum* decoctions do not exhibit any harmful effects, even when given at higher dosages of up to 2 g/kg. The absence of severe toxicity indicates that these extracts may be utilized safely for possible therapeutic applications, with little danger of substantial negative outcomes.

Additional research corroborates the findings on the antihyperglycemic properties of extracts obtained from *O. compactum* and *O. elongatum*. In a study conducted by Mancak and Çalışkan (Mancak and Çalışkan, 2023), it was shown that administering these extracts at a dosage of 400 mg/kg resulted in a substantial decrease in postprandial hyperglycemia in animal models and enhanced glucose tolerance. The test results were validated by the assessment of the area under the glucose curve, demonstrating a comparable efficacy to glibenclamide, a frequently used reference medication for managing type 2 diabetes. Furthermore, Gutiérrez et al. (Gutiérrez et al., 2022) researched to examine the impact of extracts derived from several *Origanum* species on the management of blood sugar levels in rats with experimentally induced diabetes. The study found that these extracts had significant effects in lowering blood glucose levels and improving glucose tolerance parameters. These findings suggest that these extracts have significant potential for therapeutic use in managing diabetes. The supplementary findings further support the notion that extracts derived from *Origanum* species, particularly *O. compactum* and *O. elongatum*, have the potential to be effective candidates for the creation of novel natural treatments for diabetes. Further study is required to thoroughly investigate the mechanisms of action and therapeutic potential of these substances, since they have shown the capacity to efficiently regulate postprandial glycemic response and enhance glucose tolerance.

These findings emphasize the need to do more research on these medicinal plants to get a more comprehensive understanding of their exact mode of operation and thoroughly examine their clinical capabilities. Further research might investigate the relationships between active chemicals derived from *Origanum* extracts and the metabolic pathways that regulate glucose, as well as their long-term effects on metabolic health. Gaining a deeper comprehension of these factors will enhance the development of inventive and secure therapeutic approaches for the treatment of diabetes, by using the abundant capabilities of medicinal plants like *O. compactum* and *O. elongatum*.

## 5 Conclusions

A thorough examination of the flowering tops of *O. compactum* and *O. elongatum* has revealed a significant presence of secondary metabolites, particularly phenolic compounds, which are distinguished by different profiles. *O. compactum* is characterized by high concentrations of polyphenols and flavonoids, whereas *O. elongatum* is particularly rich in salvianolic acids, luteolin, and rutin. Although the aqueous extracts of both species exhibit exceptional antioxidant capacities, the antioxidant activity of *O. compactum* is considerably superior, with a significantly higher total antioxidant capacity.

The results of antimicrobial experiments also show a more pronounced efficacy of *O. compactum* against Gram-negative bacteria, certain yeasts, and molds such as *Aspergillus niger*. Additionally, while both species display notable anticoagulant properties, *O. compactum* stands out with a slightly longer prothrombin time, suggesting a greater ability to prolong coagulation.

Although both plants possess remarkable medicinal properties, *O. compactum* emerges as overall superior in terms of antioxidant, antimicrobial, and anticoagulant efficacy. These findings highlight the significant potential of *O. compactum* for the development of innovative treatments, particularly in the fields of pharmaceuticals, nutraceuticals, and cosmetics. However, future research should focus on the molecular mechanisms underlying these biological activities, as well as on pharmacokinetic and pharmacodynamic studies of the extracts *in vivo*, while also exploring potential synergistic effects with other compounds.

## Institutional Review Board Statement

The Institutional Ethics Committee for Care and Use of Laboratory Animals of the Faculty of Sciences Dhar El Mahraz -Fez-, Sidi Mohamed Ben Abdellah University - Fez, Morocco, has reviewed and approved this study 04/2019/LBEAS.

## Funding

This research was funded by Princess Nourah bint Abdulrahman University Researchers Supporting Project number (PNURSP2024R142), Princess Nourah bint Abdulrahman University, Riyadh, Saudi Arabia.

## CRediT authorship contribution statement

**Omkulthom Al Kamaly:** Writing – review & editing, Writing – original draft, Methodology, Funding acquisition, Conceptualization. **Aziz Drioiche:** Writing – review & editing, Writing – original draft, Validation, Supervision, Methodology, Investigation, Conceptualization. **Firdaous Remok:** Software. **Soukaina Saidi:** Investigation. **Ahde El Imache:** Visualization, Data curation. **Fadoua El Makhoukhi:** Visualization, Resources. **Bshra A. Alsfouk:** Methodology, Funding acquisition, Formal analysis. **Touriya Zair:** Validation, Supervision, Project administration.

## Declaration of Competing Interest

The authors declare that they have no known competing financial interests or personal relationships that could have appeared to influence the work reported in this paper.
